# BRAID: Input-driven nonlinear dynamical modeling of neural-behavioral data

**Published:** 2025-09-23

**Authors:** Parsa Vahidi, Omid G. Sani, Maryam M. Shanechi

**Affiliations:** 1 Electrical and Computer Engineering, University of Southern California (USC), Los Angeles, CA; 2 Computer Science, University of Southern California (USC), Los Angeles, CA; 3 Biomedical Engineering Viterbi School of Engineering, University of Southern California (USC), Los Angeles, CA

## Abstract

Neural populations exhibit complex recurrent structures that drive behavior, while continuously receiving and integrating external inputs from sensory stimuli, upstream regions, and neurostimulation. However, neural populations are often modeled as autonomous dynamical systems, with little consideration given to the influence of external inputs that shape the population activity and behavioral outcomes. Here, we introduce BRAID, a deep learning framework that models nonlinear neural dynamics underlying behavior while explicitly incorporating any measured external inputs. Our method disentangles intrinsic recurrent neural population dynamics from the effects of inputs by including a forecasting objective within input-driven recurrent neural networks. BRAID further prioritizes the learning of intrinsic dynamics that are related to a behavior of interest by using a multi-stage optimization scheme. We validate BRAID with nonlinear simulations, showing that it can accurately learn the intrinsic dynamics shared between neural and behavioral modalities. We then apply BRAID to motor cortical activity recorded during a motor task and demonstrate that our method more accurately fits the neural-behavioral data by incorporating measured sensory stimuli into the model and improves the forecasting of neural-behavioral data compared with various baseline methods, whether input-driven or not.

## 1 Introduction

Understanding the relationship between neural activity and behavior is a critical goal in neuroscience and neurotechnology. Neural activity and its temporal structure, or *“dynamics”*, during a behavior are formed by the interplay between (1) the recurrent networks within a brain area, i.e., *intrinsic dynamics*, and the (2) temporally-structured inputs it receives during the behavior (Remington et al., 2018; Vyas et al., 2020). A neural population may receive inputs from measurable sources such as sensory stimuli, electrical/optogenetic neurostimulation (Buonomano & Maass, 2009; Seely et al., 2016; Susilaradeya et al., 2019; Sauerbrei et al., 2020; Shenoy & Kao, 2021; Yang et al., 2021b; Vahidi et al., 2024), as well as from other upstream brain areas (Sauerbrei et al., 2020; Shenoy & Kao, 2021), which could be included in multi-regional recordings (Jun et al., 2017; Steinmetz et al., 2019). However, even easily measurable external inputs (e.g., sensory stimuli) are often not explicitly considered when modeling neural-behavioral activity, which can lead to a conflation of intrinsic and input-driven contributions, creating challenges for interpretation (Seely et al., 2016; Sauerbrei et al., 2020; Vahidi et al., 2024). Beyond disentangling intrinsic dynamics from input dynamics, incorporating measured inputs into models can also enhance the behavior decoding performance in neurotechnologies such as stimulation-based closed-loop controllers (Yang et al., 2018; 2021b).

Another challenge is to disentangle neural dynamics that are relevant to a specific behavior from other neural dynamics, and to prioritize the former. This is critical as the majority of neural variance may not be relevant to the behavior of interest (Churchland et al., 2012; Mante et al., 2013; Kobak et al., 2016; Allen et al., 2019; Engel & Steinmetz, 2019; Stringer et al., 2019; Sani et al., 2021). While most prior works use unsupervised methods when modeling neural activity as latent variable dynamical systems (Aghagolzadeh & Truccolo, 2015; Gao et al., 2016; Wu et al., 2017; Pandarinath et al., 2018; Hernandez et al., 2020; Rutten et al., 2020; Kim et al., 2021), recent works have shown improved learning of behaviorally relevant neural dynamics by using behavior data during learning in a supervised manner (Sani et al., 2021; Hurwitz et al., 2021; Kramer et al., 2022; Gondur et al., 2024; Vahidi et al., 2024; Abbaspourazad et al., 2024; Sani et al., 2024; Oganesian et al., 2024).

Yet another challenge is posed by the nonlinearities in neural-behavioral data. While linear models have been extremely effective in approximating neural dynamics (Hastie et al., 2009; Churchland et al., 2012; Mante et al., 2013; Cunningham & Yu, 2014; Kao et al., 2015; Kobak et al., 2016; Abbaspourazad et al., 2021; Sani et al., 2021), they may require higher dimensional latent representations compared to nonlinear models (Yang et al., 2019; Nozari et al., 2024; Sani et al., 2024), and do not provide interpretability for nonlinear dynamical phenomena such as multi-stable fixed points and limit cycles (Kim et al., 2021; Durstewitz et al., 2023). Moreover, unlike linear models, for nonlinear models the relationship between the intrinsic dynamics and an inference model that is fitted to estimate the latent states from observations is not analytically known, posing a challenge for studying intrinsic dynamics (see section 3.1).

Here, we address all aforementioned challenges by introducing Behaviorally Relevant Analysis of Intrinsic Dynamics (BRAID), a new method with the following key contributions. *First*, BRAID captures complex nonlinear structures in neural-behavioral-input data, offering greater expressivity than linear methods. *Second*, by optimizing multi-step-ahead forecasts of neural-behavior data, BRAID simultaneously learns two representations for neural dynamics: the predictor and the generative form representations (see section 3.1), the latter of which describes intrinsic dynamics. *Third*, by explicitly modeling the influence of measured inputs, BRAID disentangles their dynamics from intrinsic dynamics to more closely reflect the neuronal networks within the recorded brain region. *Fourth*, we introduce a multi-stage learning framework that dissociates and prioritizes the learning of intrinsic behaviorally relevant neural dynamics, while considering measured inputs (see section 3.2). *Fifth*, we introduce additional preprocessing and post-hoc learning stages that allow behavior-specific dynamics to be dissociated from behaviorally relevant neural dynamics (see section 3.3).

We validate BRAID in multiple simulated datasets with distinct nonlinear structures and show its capability to accurately learn the underlying nonlinear model, resulting in an interpretable representation of intrinsic dynamics. We then apply our method to electrophysiological data recorded from a non-human-primate (NHP) performing sequential reaches (O’Doherty et al., 2017). Our results indicate that accounting for both nonlinearity and sensory inputs improves neural-behavioral prediction, suggesting a more accurate representation of intrinsic behaviorally relevant neural dynamics.

## 2 Related Work

Our work addresses multiple problems simultaneously, which makes it related to various methods that tackle a subset of these problems. A summary of related methods is provided in table 1.

First, a key ability of BRAID is to incorporate measured inputs to disentangle intrinsic dynamics from input dynamics. Other nonlinear modeling methods (Gao et al., 2016; Sussillo et al., 2016; Wu et al., 2017; Pandarinath et al., 2018; Rutten et al., 2020; Hurwitz et al., 2021; Kim et al., 2021; Abbaspourazad et al., 2024; Sani et al., 2024) have not addressed modeling measured external inputs and their impact on neural-behavioral data. As demonstrated by Vahidi et al. (2024), not considering external inputs can lead to the dynamics of these inputs being misinterpreted as intrinsic neural dynamics. To overcome this challenge, Vahidi et al. (2024) introduce a linear dynamical modeling method, termed IPSID, which explicitly incorporates measured external inputs into the model. However, IPSID is an analytical, and strictly linear method that cannot capture nonlinearities. By incorporating the strengths of this linear modeling work into BRAID, we can account for measured external inputs and dissociate their dynamics while allowing every model element to be nonlinear. We use IPSID as a key baseline to show the benefit of enabling nonlinearity in our method (see appendix A.2 for details). We also show the results for the special case of setting all model elements as linear in our method (referred to as linear BRAID), which fits in a linear model similar to IPSID.

Second, a key capability of BRAID is that it dissociates behaviorally relevant neural dynamics into a distinct part of the latent states and prioritizes their learning, while also being able to learn neural-specific and behavior-specific dynamics using additional latent states. Among prior works, two recent nonlinear methods termed DPAD (Sani et al., 2024) and TNDM (Hurwitz et al., 2021) aim to dissociate behaviorally relevant dynamics from other neural dynamics, but neither method dissociates the third category of dynamics, i.e., the behavior-specific dynamics. More importantly, neither DPAD nor TNDM incorporates external inputs into the model to dissociate intrinsic dynamics from input dynamics. Finally, DPAD learns models based on 1-step-ahead prediction of neural-behavioral data and does not explicitly learn the intrinsic dynamics, whereas BRAID adds m-step-ahead predictions into the loss to optimize forecasting and also explicitly learns a generative representation of intrinsic dynamics. TNDM on the other hand is a sequential autoencoder (similar to LFADS, Pandarinath et al., 2018), i.e., it optimizes reconstruction of a window of data after ingesting the entire window as input. We compared our results with both DPAD and TNDM, although DPAD’s architecture is closer to ours. In fact, the comparisons with DPAD can also be thought of as ablation studies that show the benefit of incorporating external inputs and forecasting in our method.

Third, we learn behaviorally relevant neural dynamics, or in other words the shared neural-behavioral dynamics, in an initial optimization focused on learning these dynamics, while leaving the learning of other neural dynamics to a separate subsequent optimization. This approach, prioritizes behaviorally relevant neural dynamics in the sense that we can fit models with low dimensional latent states that are purely focused on these dynamics (Sani et al., 2021). Besides DPAD (Sani et al., 2024), a few other works, including TNDM, propose nonlinear approaches for learning dynamics shared between two modalities (Hurwitz et al., 2021; Kramer et al., 2022; Gondur et al., 2024). However, these works use a combined loss to optimize the reconstruction of both modalities in the same optimization. While this approach can capture the dynamics shared between modalities, it does not prioritize them over dynamics specific to either modality (Sani et al., 2024). Moreover, most multi-modal approaches do not model the effect of external inputs (Hurwitz et al., 2021; Gondur et al., 2024). One multi-modal model, termed mmPLRNN (Kramer et al., 2022), which models dynamics of two modalities with a piecewise-linear RNN (see appendix A.2 for details), supports modeling the effect of external inputs, although this capability was not demonstrated in Kramer et al. (2022). Nevertheless, we include comparisons with mmPLRNN with input as one baseline. Finally, as another ablation study to assess the importance of prioritizing behaviorally relevant dynamics, we also implement an unsupervised version of BRAID, termed U-BRAID, that removes the behaviorally relevant optimization step and instead learns all neural dynamics in one optimization step while still incorporating external inputs into the model (see section 3 and appendix A.2 for details).

Most other prior nonlinear methods only consider neural signals during modeling without considering behavior or external inputs (Gao et al., 2016; Pandarinath et al., 2018; Hernandez et al., 2020; Rutten et al., 2020; Kim et al., 2021) or do not use dynamic models (Zhou & Wei, 2020; Schneider et al., 2023) and thus are vastly different from our method. Nevertheless, we include comparisons with LFADS (Pandarinath et al., 2018) and CEBRA (Schneider et al., 2023) as additional baselines. We list the differences of some of these methods with our method in table 1.

## 3 Methods

### 3.1 BRAID Model

We model the neural activity $\left(y_{k}\in {\mathbb{R}}^{n_{y}}\right)$ and behavior $\left(z_{k}\in {\mathbb{R}}^{n_{z}}\right)$ as observations of a nonlinear dynamical system that is driven by measured $\left(u_{k}\in {\mathbb{R}}^{n_{u}}\right)$ and/or unmeasured inputs $\left(w_{k}\in {\mathbb{R}}^{n_{x}}\right)$:

(1) $$\left\{\begin{array}{lll} x_{k+1}^{s} & = & A_{fw}\left(x_{k}^{s}\right)+K_{fw}\left(u_{k}\right)+w_{k}\\ y_{k} & = & C_{y}\left(x_{k}^{s},u_{k}\right)+v_{k}\\ z_{k} & = & C_{z}\left(x_{k}^{s},u_{k}\right)+\epsilon_{k} \end{array}\right.$$

Here, $x_{k}^{s}\in {\mathbb{R}}^{n_{x}}$ represent the latent states of the system and evolve according to intrinsic dynamics $A_{fw}.v_{k}\in {\mathbb{R}}^{n_{y}}$ and $\epsilon_{k}\in {\mathbb{R}}^{n_{z}}$ represent observation noises. Given this dynamical system, one can recursively infer the latent state from neural observations $y_{k}$ using an RNN as follows

(2) $$x_{k+1|k}=A\left(x_{k|k-1}\right)+K\left(y_{k},u_{k}\right)$$

where $x_{k+1|k}$ (or simply $x_{k+1}$) is defined as the inferred latent state based on $\left\{y_{1},\ldots ,y_{k}\right\}$ and $\left\{u_{1},\ldots ,u_{k}\right\}$. Given the latent nature of the states, even when inference is optimal (e.g., in a Kalman filter), the inferred states will *not* be equal to the internal states $x_{k}^{s}$ in equation 1 (Katayama, 2006), which is why we use different notations for the states in equations 1 and 2. More importantly, note that A and K in equation 2 are distinct from $A_{fw}$ and $K_{fw}$ in equation 1. This is because A and K represent the “predictor form” representation of dynamics, describing how the inferred latent state recursively evolves over time as samples of $y_{k}$ and $u_{k}$ are observed, whereas $A_{fw}$ and $K_{fw}$ represent the “generative form” representation of dynamics that describe how the latent states themselves evolve, purely based on their *intrinsic dynamics* – so $A_{fw}$ is what ultimately describes the intrinsic dynamics. For linear systems, there is an analytical bidirectional relationship between predictor and generative form representations (defined by the Kalman filter, see Katayama, 2006), whereas for nonlinear systems in general, this relationship is not known. Thus, we devise an approach that allows us to learn both representations of dynamics from data.

**Figure 1:**
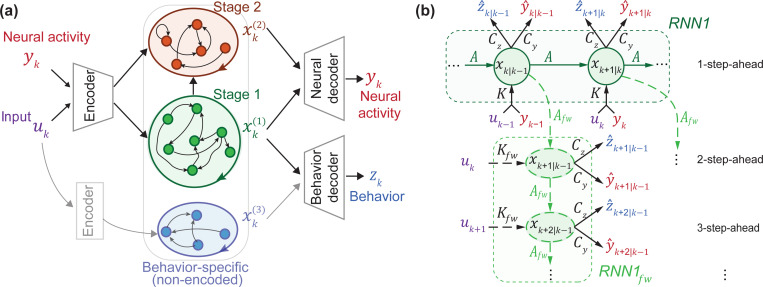
BRAID model architecture. **(a)** BRAID dissociates the dynamics of neural-behavioral data into three latent states $x_{k}^{\left(1\right)}$, $x_{k}^{\left(2\right)}$, and $x_{k}^{\left(3\right)}$: 1) the dynamics shared between neural and behavioral modalities (learned in stage 1 by RNN1 and $RNN1_{fw}$), 2) any remaining dynamics private to neural activity (learned in stage 2 by RNN2 and $RNN2_{fw}$), and 3) Input-driven, behavior-specific dynamics not encoded in neural activity (learned per section 3.3 by RNN3 and $RNN3_{fw}$). (b) For each latent state, we simultaneously learn a predictor and generative form representation of the dynamics (denoted by A and $A_{fw}$), by optimizing m-step-ahead prediction of neural-behavioral data. This interconnected two-RNN system is visualized for RNN1 and $RNN1_{fw}$ in this computation graph. The superscript $\cdot^{\left(1\right)}$ indicating that parameters are for RNN1 and $RNN1_{fw}$ is omitted for simplicity.

Critically, to predict the latent state (or neural-behavioral data) multiple (m>1) steps into the future without new neural observations and using only new inputs $u_{k}$, we need to propagate the latent state ahead according to its *intrinsic* dynamics, i.e., the “generative form” representation of dynamics, as

(3) $$x_{k+m|k}=A_{fw}\left(x_{k+m-1|k}\right)+K_{fw}\left(u_{k+m-1}\right)$$

where $x_{k+m|k}$ denotes the latent state at time step k+m, generated given $\left\{y_{1},\ldots ,y_{k}\right\}$ and $\left\{u_{1},\ldots ,u_{k+m-1}\right\}$. Note that for m=2, the right hand side of equation 3 would have $x_{k+1|k}$, which is given by equation 2. Thus, m-step-ahead inference of the latent state engages both the predictor and generative form representations of the dynamics via equations 2 and 3, respectively. As an alternative interpretation, the m-step-ahead prediction of the latent state (or neural or behavioral data), for m>1, involves two RNNs operating in complementary fashion (figure 1b):

1. The first RNN (RNN, parameterized by A and K) takes in neural and input time series and recursively estimates the 1-step-ahead prediction $\left(x_{k|k-1}\right)$.
2. The second RNN ($RNN_{fw}$, parameterized by $A_{fw}$ and $K_{fw}$) takes in the 1-step-ahead predicted state from the first RNN and propagates it m−1 additional steps ahead according to the intrinsic latent dynamics of the model, to get the m-step-ahead predictions ($x_{k+m-1|k-1}$, for m>1).

Overall, the BRAID model is comprised of six distinct transformations: A(⋅), $A_{fw}(\cdot )$, K(⋅), $K_{fw}(\cdot )$, $C_{z}(\cdot )$, and $C_{y}(\cdot )$. $A/A_{fw}$ describe predictor/generative form recursions of the latent state. $K/K_{fw}$ describe predictor/generative form encoders. $C_{z}$ and $C_{y}$ describe behavior and neural decoders. We implement these six transformations as multi-layer perceptrons (MLPs) with arbitrary user-specified number of units and hidden layers. As a special case, any (or all) of these mappings can be replaced by a linear mapping (i.e., an MLP with no hidden layer and a linear activation).

We learn the parameters specifying all six transformations of the model by optimizing a weighted sum of m-step-ahead neural-behavioral prediction errors (for $m\in \left[m_{1},m_{2},\ldots ,m_{L}\right]$) as our losses

(4) $$L_{z}=\sum_{i=1}^{L}\alpha_{z_{m_{i}}}\text{MSE}\left(z_{k+m_{i}},C_{z}\left(x_{k+m_{i}|k},u_{k+m_{i}}\right)\right)\;L_{y}=\sum_{i=1}^{L}\alpha_{y_{m_{i}}}\text{MSE}\left(y_{k+m_{i}},C_{y}\left(x_{k+m_{i}|k},u_{k+m_{i}}\right)\right)$$

where MSE(·) indicates the mean-squared error loss, L denotes the number of steps ahead simultaneously included in the loss, and $\alpha_{z_{m_{i}}}$ and $\alpha_{y_{m_{i}}}$ denote the weights used in the sum. In this work, we always set $\alpha_{z_{m_{i}}}$ and $\alpha_{y_{m_{i}}}$ to 1. Moreover, although the decoders in BRAID can optionally take both the latent state and the external input $u_{k}$ (lines 2–3 of equation 1), in our real data analyses we do not provide $u_{k}$ to decoders and generate predictions only based on the latent states.

### 3.2 Prioritization of behaviorally relevant over other neural dynamics

To dissociate behaviorally relevant neural dynamics from other neural dynamics and prioritize the former, we break the latent state $x_{k}$ into two sections ($x_{k}^{\left(1\right)}$ and $x_{k}^{\left(2\right)}$) and learn these two sections in two learning stages. We denote the model parameters associated with each model section using a $\cdot^{\left(1\right)}$ or $\cdot^{\left(2\right)}$ superscript, e.g., $A^{\left(1\right)}$ and $A^{\left(2\right)}$. We provide the full two-section formulation for the model in appendix A.1.1 and the optimization details in appendix A.1.2. Briefly, each of the two learning stages consist of 2 optimizations, as follows:

**Stage 1:** Learning RNN1 and $RNN1_{fw}$

1a Learn $A^{\left(1\right)}$, $A_{fw}^{\left(1\right)}$, $K^{\left(1\right)}$, $K_{fw}^{\left(1\right)}$ and $C_{z}^{\left(1\right)}$, and extract latent states $x_{k}^{\left(1\right)}$ and $x_{k+m|k}^{\left(1\right)}$ (for m>1) by minimizing the behavior prediction loss $L_{z}$ from equation 4.

1b Learn $C_{y}^{\left(1\right)}$ by predicting neural data from $x_{k}^{\left(1\right)}$ and $x_{k+m|k}^{\left(1\right)}$, while minimizing the neural prediction loss $L_{y}$ from equation 4.

**Stage 2:** Learning RNN2 and $RNN2_{fw}$

2a Learn $A^{\left(2\right)}$, $A_{fw}^{\left(2\right)}$, $K^{\left(2\right)}$, $K_{fw}^{\left(2\right)}$, and $C_{y}^{\left(2\right)}$, and extract latent states $x_{k}^{\left(2\right)}$ and $x_{k+m|k}^{\left(2\right)}$ (for m>1) by minimizing the neural loss $L_{y}$, while including outputs of stage 1b as part of the predictions.

2b Learn $C_{z}^{\left(2\right)}$ by predicting behavior from $x_{k}^{\left(2\right)}$ and $x_{k+m|k}^{\left(2\right)}$, while minimizing the behavior loss $L_{z}$ from equation 4, and including outputs of stage 1a as part of the predictions.

The explicit dissociation of the relevant dynamics and the above two-stage optimization allow us to first preferentially learn the (low-dimensional) shared dynamics between the two observations, i.e., the behaviorally relevant neural dynamics $\left(x_{k}^{\left(1\right)}\right)$, in stage 1. Then in stage 2, we learn any residual neural dynamics $\left(x_{k}^{\left(2\right)}\right)$, which, as depicted in figure 1a, can depend on the behaviorally relevant dynamic (see appendix A.1 for details). The optional stage 2 is of interest for explaining neural dynamics beyond the ones related to behavior. This multi-stage approach has similarities to Sani et al. (2024), but here we have: 1) additional signals $\left(u_{k}\right)$, 2) different losses, 3) a forecasting RNN within each model section (figure 1b), and additional steps that are discussed in the next section.

### 3.3 Dissociation of behavior-specific dynamics

Optimizing behavior prediction (stage 1a) given neural activity $y_{k}$ and input $u_{k}$ can lead to learning behavior dynamics that are predictable from the input but are not encoded in the recorded neural activity. Although learning such behavior-specific dynamics enhances behavior decoding, it poses an interpretation challenge for neuroscience applications because one would not know what part of the learned dynamics are represented in the recorded brain regions. As shown in Vahidi et al. (2024), this may lead to a misinterpretation of input-driven behavior-specific dynamics as intrinsic dynamics of the recorded brain region. To mitigate this possibility, we develop two additional steps in our method, that can 1) exclude such behavior-specific dynamics from $x_{k}^{\left(1\right)}$, and 2) learn them separately as a distinct latent state $x_{k}^{\left(3\right)}$ (figure 1a). Details are provided in appendix A.1.3. Briefly, *first*, to exclude behavior-specific dynamics, we introduce an optional preprocessing stage that predicts behavior from neural data, and passes this neurally-predicted behavior to be used in stages 1a and 2b. This preprocessing step ensures that the behaviorally relevant states learned in stage 1 $\left(x_{k}^{\left(1\right)}\right)$ are encoded in recorded neural activity $y_{k}$, which can be crucial for interpretability in neuroscience studies. In our analyses of the real datasets (section 4.2), we always include this preprocessing step. *Second*, to still be able to learn behavior-specific dynamics, we add an optional post-hoc learning step (i.e., stage 3) that fits RNN3 and $RNN3_{fw}$ to any unexplained behavior and learns these input-driven behavior-specific dynamics as a distinct latent state $x_{k}^{\left(3\right)}$. As we show in simulations (see section A.5.1 and figure A.2), the preprocessing step can exclude non-encoded behavior dynamics. When desired in an application, the optional stage 3 can learn such dynamics to offset any behavior decoding loss incurred due to the preprocessing step, while still maintaining the interpretability of the model. We did not apply this post-hoc step in our real data analyses (section 4.2).

### 3.4 Inference and Evaluation Metrics

After learning BRAID’s parameters, we can readily use the learned mappings A, K (and $A_{fw}$ and $K_{fw}$) to infer the 1-(and multi)-step-ahead predicted states $x_{k}$ (and $x_{k+m|k}$) using equations 2 (and 3) for the held-out test data. Predicted neural activity and behavior are obtained by applying their corresponding decoders $C_{y}$ and $C_{z}$ to these inferred states. We also use the term “decoding” for behavior predictions because our model predicts behavior only using neural data and inputs, and never using behavior itself. To evaluate the performance of our models, we perform 5- and 2-fold cross-validation, for real data and simulation analyses, respectively. We report Pearson’s Correlation Coefficient (CC) and in some cases (see table A.8) also the coefficient of determination $\left(R^{2}\right)$ between the predicted and actual observation, averaged over dimensions. We further report the m-step-ahead prediction accuracy, which reflects how well the intrinsic dynamics $A_{fw}$ are learned. For simulation analyses with linear recursions $\left(A_{fw}\right)$, we additionally evaluate the learned intrinsic dynamics by comparing the eigenvalues of $A_{fw}$ between the true and learned model (see appendix A.1.5).

## 4 Experimental Results

### 4.1 Simulation Experiments

We validated BRAID in three simulations with different nonlinear neural-behavioral-input structures to show that it can learn intrinsic behaviorally relevant neural dynamics in presence of inputs.

#### 4.1.1 BRAID achieves near optimal neural-behavioral predictive accuracy in nonlinear input-driven simulations

**Figure 2:**
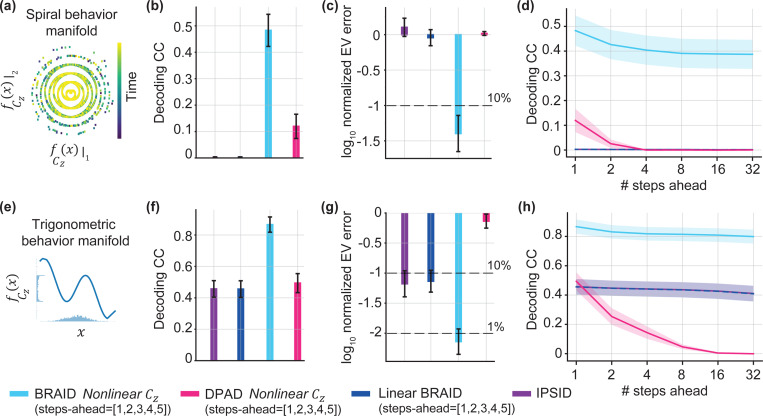
BRAID better learns the intrinsic shared dynamics by simultaneously modeling input and nonlinearity, and by optimizing forecasting. **(a-d)** Results for simulations with a spiral behavioral manifold. **(b)** 1-step-ahead behavior decoding for nonlinear BRAID, nonlinear DPAD, linear BRAID, and IPSID **(c)**. Error in identifying intrinsic dynamics of the true model quantified by the error in learning the eigenvalues of $A_{fw}$. **(d)** Behavior decoding forecasts for 1 to 32 steps ahead, enabled by learning the intrinsic dynamics $\left(A_{fw}\right)$, with predictions optimized for [1,2,3,4,5]-steps-ahead (section 3). **(e-h)** Same as (a-d) for simulations with a trigonometric behavior mapping.

First, we considered an input-driven dynamical system as in equation 1, but with only the behavior mapping $C_{z}$ being nonlinear with the mapping $f_{C_{z}}(\nu ):=a\sin(\nu )+b\nu$, as detailed in section A.4.2 (figure 2a). We generated 10 random parameter sets as our true models and generated data from them. First, we implemented an automatic selection of nonlinearity for BRAID by setting each of A, K, $C_{y}$, or $C_{z}$ to linear or nonlinear, resulting in 2^4^ different BRAID models, and finding the model with the best behavior decoding in the training data. Across all 10 realizations and 2 cross-validated folds, setting the behavior decoder $C_{z}$ to be nonlinear was correctly identified as the best performing nonlinearity in 100% of the cases. Additionally, we evaluated BRAID with nonlinearity only in one of A, K, $C_{y}$, or $C_{z}$. The model with nonlinear behavior decoder $C_{z}$ outperformed other nonlinearity choices as well as linear models (i.e., IPSID and the fully linear BRAID) in behavior decoding and neural prediction. BRAID further outperformed DPAD, which is nonlinear but does not account for the input $u_{k}$, in neural-behavioral prediction. In fact, both BRAID with nonlinear $C_{z}$ and BRAID with automatic nonlinearity selection achieved almost the same neural-behavioral prediction as the true simulated models, demonstrating BRAID’s success in accurately learning the nonlinear input-driven dynamical system (table 2).

#### 4.1.2 BRAID dissociates intrinsic dynamics from input dynamics in simulations

**Figure 3:**
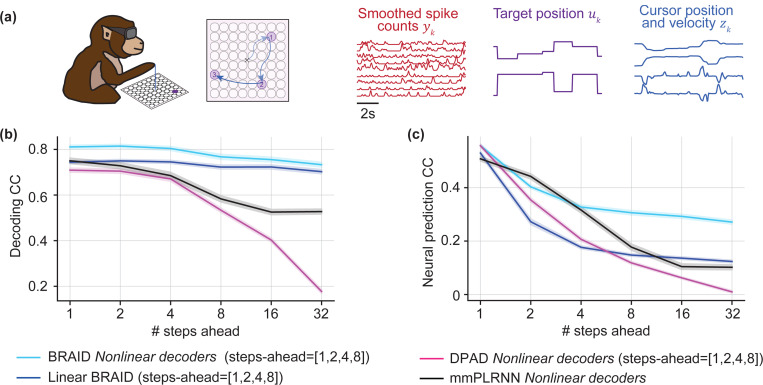
BRAID outperforms baselines in neural-behavioral forecasting. **(a)** Dataset and task visualization. **(b, c)** Behavior and neural activity forecasting correlation coefficient (CC) for BRAID, linear BRAID, DPAD, and mmPLRNN at low state dimension regime ($n_{x}=16$). Shaded areas show the s.e.m. across the 7 recording sessions and 5 cross-validation folds.

Next, we sought to validate BRAID’s ability to disentangle intrinsic and input-driven contributions to neural dynamics. BRAID simultaneously learns a predictor form and a generative form representation of the dynamics, the latter of which directly describes the intrinsic dynamics in terms of the mapping $A_{fw}(\cdot )$ (section 3.1, figure 1b). In this simulation, we kept the ground truth state transitions linear so that we could precisely quantify the intrinsic dynamics and their learning error via the eigenvalues of the state transition matrix $A_{fw}$ (appendix A.1.5). We analyzed data from three sets of simulated dynamical systems with distinct nonlinear structures (see appendix A.4 for details): (1) Spiral behavior manifold (figure 2a-d), (2) trigonometric behavior manifold (also explained in section 4.1.1, figure 2e-h), and (3) trigonometric input-encoder (figure A.1). For each simulation, we generated realizations from 10 different systems with randomly generated sets of parameters. Across all three simulations, BRAID accurately learned the intrinsic dynamics, resulting in smaller error in eigenvalues of the transition matrix compared with DPAD, which is nonlinear but does not consider the input, and compared with linear BRAID and IPSID, which consider input but are linear (figures 2c,g, A.1d). We demonstrate in an ablation study that learning a separate generative model for forecasting is crucial for correct learning of the intrinsic dynamics (table A.3). These more accurate intrinsic dynamics coupled with the input also resulted in BRAID achieving better behavior decoding (figures 2b,f, A.1a) and neural prediction (figure A.1c) compared to baselines. These results suggest that failing to account for either nonlinearity or input may lead to less accurate models and a misinterpretation of the intrinsic dynamics in nonlinear data.

Another metric for how well intrinsic dynamics are learned is forecasting, where behavior is predicted multiple steps into the future, without observing new neural data and only by observing the future input (section 3.1). Forecasting evolves the state dynamics according to the learned intrinsic dynamics (equation 3) and thus validates their accurate learning. We performed forecasting up to 32 steps ahead and found that the nonlinear models with input consistently outperformed linear models as well as the nonlinear DPAD, which does not consider input (figures 2d,h and A.1e,f).

### 4.2 Non-human primate motor cortical activity during reaching

We applied our method to a publicly available dataset recorded from a non-human primate (NHP) performing reaching movements O’Doherty et al. (2017) (figure 3a). We took either the smoothed spike counts or raw LFP from primary motor cortex (M1) as neural time-series $y_{k}$, fingertip’s position and velocity as behavior $z_{k}$, and sensory task instructions (target location) as the input $u_{k}$ (appendix A.3). Sensory inputs can have their own dynamics, which are distinct from the intrinsic dynamics of the motor cortex. Our goal is to learn the intrinsic dynamics in M1 related to movement while disentangling them from the dynamics of sensory input and also from any behavior-specific dynamics. Therefore, we importantly include BRAID’s behavior preprocessing stage (section 3.3).

We fitted BRAID with different nonlinearity choices as in our simulations: (1) nonlinear recursion $A(\cdot )/A_{fw}(\cdot )$, (2) nonlinear encoder $K(\cdot )/K_{fw}(\cdot )$, (3) nonlinear decoders $C_{y}(\cdot )$ and $C_{z}(\cdot )$, and a fully linear variant, linear BRAID. We included m=[1,2,4,8]-steps-ahead predictions in the BRAID loss, which for m>1 engage the intrinsic behaviorally relevant dynamics $\left(A_{fw}\right)$ and allow their learning. To evaluate the learned intrinsic dynamics, we report neural-behavioral forecasting accuracy for different step-ahead horizons (figures 3b-c, and A.3 for LFP), while tabulating the 4steps-ahead (i.e., 200ms) results to highlight BRAID’s advantage in forecasting (tables 3 and A.4). In addition to these results in the low-dimensional regime (stage 1 only, $n_{x}=n_{1}=16$), we also report the results in the high-dimensional regime (both stages, $n_{x}=64$, $n_{1}=16$) (table 3, figures A.5 and A.6). Among nonlinearity configurations, BRAID with nonlinear decoders provided the best fit to neural-behavioral data (table A.4). Moreover, BRAID’s behavior forecasting performance improved as more neurons were included (table A.7).

Next, we compared BRAID’s neural-behavioral forecasting to several ablation baselines (table 3 and figures 3 and A.5). First, BRAID outperformed linear BRAID, i.e., a similar but fully linear model. Second, BRAID outperformed DPAD (Sani et al., 2024), which can have decoder nonlinearities but does not consider inputs. BRAID’s advantage shows the importance of considering the effects of sensory inputs on neural-behavioral dynamics. Third, we compared to U-BRAID, which removes the first stage of BRAID and thus loses prioritization. BRAID consistently outperformed U-BRAID in behavioral forecasting, but did not match the neural forecasting of U-BRAID unless it was given enough latent state dimensions (table 3, $n_{x}=64$), which is expected given U-BRAID’s singular objective being neural prediction. This comparison shows the benefit of prioritization for learning low-dimensional representations of intrinsic behaviorally relevant dynamics, and confirms that with its stage 2, BRAID can capture any remaining non-behavioral neural dynamics. Finally, BRAID’s low dimensional latent state trajectories were better separated for different movement directions compared to those of U-BRAID and DPAD, suggesting that BRAID’s latent states are more congruent with behavior, i.e., more behaviorally relevant (figure A.4).

We also compared BRAID’s neural-behavioral forecasting performance to mmPLRNN, which models multi-modal data using piecewise-linear RNNs, and has the option to model inputs although prior work had not explored this input option. BRAID outperformed input-driven mmPLRNN networks in forecasting both behavior and neural activity, across all forecasting horizons, with the exception of 2-steps-ahead neural prediction (table 3, figure 3). Note that BRAID’s better decoding is achieved despite the fact that mmPLRNN, by design, incorporates behavior *as an input* during inference, whereas BRAID and DPAD do not. Also, we compared BRAID to TNDM (Hurwitz et al., 2021), a nonlinear sequential autoencoder that models neural-behavioral dynamics, but does not include the effect of external inputs. We extended TNDM beyond the original work to create a version that also adds sensory inputs as an additional input besides neural activity (appendix A.2.6). We analyzed non-smoothed spike counts from the same dataset and found that BRAID significantly outperformed TNDM in behavior decoding while achieving comparable neural prediction (table A.5). Extending TNDM to include inputs significantly improved its behavior decoding, but it still did not reach that of BRAID (table A.5). Finally, we compared BRAID with CEBRA (Schneider et al., 2023), which is a non-dynamic convolutional encoder with a contrastive loss on behavior (section A.2.8), and with LFADS (Pandarinath et al., 2018), which is an unsupervised sequential autoencoder (section A.2.7). BRAID outperformed both these baselines in neural-behavioral prediction (table A.6).

## 5 Discussion

We introduced BRAID, a method for input-driven nonlinear dynamical modeling that disentangles intrinsic shared dynamics between two observation modalities from the effect of input. Here, we assume some external inputs are measured (e.g., from sensory stimuli or other brain regions) and are available for modeling. This approach is distinct from the input-inference approach, where unmeasured inputs are inferred from measured neural activity (Pandarinath et al., 2018; Schimel et al., 2022). These two approaches are in a sense complementary. In our approach, any measured inputs can be explicitly incorporated into the model to dissociate their dynamics from the intrinsic dynamics of the measured neural-behavioral data. Practically, one cannot measure all inputs to a given brain area, so our approach does not rule out the influence of unmeasured inputs on the learned intrinsic dynamics. One could thus use the input-inference approach to infer such unmeasured inputs from all measured signals. Note, however, that inferred inputs are ultimately a function of measured signals and thus do not add any new information (unlike measured inputs); rather they can be thought of as a decomposition of the measured signals based on certain assumptions (e.g., smoothness).

While BRAID’s learning is supervised by behavior, it only uses neural activity and input (*not* behavior) during inference. This supervision allows stage 1 to extract behaviorally relevant intrinsic dynamics with priority, while later stages learn neural-specific or behavior-specific dynamics in independent optimizations. This multi-stage learning has similarities to some prior works (Sani et al., 2021; Vahidi et al., 2024; Sani et al., 2024; Oganesian et al., 2024), but is fundamentally different from other works that use a single multi-modal optimization loss (Kramer et al., 2022; Gondur et al., 2024; Hurwitz et al., 2021), which may miss prioritization of shared dynamics over unshared dynamics (Sani et al., 2024). Some of the latter group are indeed focused on multi-modal inference and fuse all shared and unshared information into the same latent space (Kramer et al., 2022; Gondur et al., 2024). Multi-stage methods avoid this fusion by focusing on shared dynamics during a first optimization stage with only cross-modality prediction (e.g., behavior decoding) as the objective.

To disentangle input dynamics from intrinsic dynamic, BRAID consists of three stages, each learning a predictor and a generator model. In each stage, BRAID’s predictor model in that stage (e.g., RNN1) infers the latent states, which are subsequently employed as the input to compute m-step-ahead predictions via the associated generative model (e.g., $RNN1_{fw}$). BRAID’s predictor and generative models are learned jointly to maximize the m-step-ahead log-likelihood. This has analogies to the encoder-decoder architectures such as those commonly used in variational inference (Kingma & Welling, 2013). Specifically, the predictor and generator RNNs in BRAID have roles similar to those of the encoder and decoder in variational inference, respectively. However, in variational inference, the posterior distribution of the unobserved variables given the data is parametrized and a part of the optimization loss aims to enforce that distribution on the inferred latent variables (Chung et al., 2015; Krishnan et al., 2015; Fraccaro et al., 2016; Luk et al., 2024). In contrast, we do not impose such parametrization on the latent states in BRAID. Developing variational methods with the same multi-section architecture and multi-stage learning as BRAID is an interesting future direction.

Similar to many prior works including some in neuroscience (Abbaspourazad et al., 2024; Sani et al., 2024; Chung et al., 2015; Krishnan et al., 2015; Fraccaro et al., 2016; Luk et al., 2024), BRAID has a causal formulation and performs inference by recursively inferring the next latent state after each new observation. This is distinct from some nonlinear methods in neuroscience (Gao et al., 2016; Pandarinath et al., 2018; Hernandez et al., 2020; Hurwitz et al., 2021; Keshtkaran et al., 2022; Gondur et al., 2024; Karniol-Tambour et al., 2024) that perform inference non-causally in time. Thus, BRAID may be useful for real-time decoding in brain-computer interfaces (Shanechi, 2019).

BRAID, as presented, is designed for single-session settings. Extending it for cross-session generalization is an interesting future direction that can follow approaches used in the literature, such as aligning neural manifolds across sessions (Farshchian et al., 2019), adaptive modeling approaches (Ahmadipour et al., 2021; Yang et al., 2021a), or incorporating session-specific read-in and read-out mappings while keeping shared model parameters fixed (Pandarinath et al., 2018). The latter allows training across multiple sessions and adapting to new sessions with minimal data by learning session-specific matrices. Furthermore, BRAID’s ability to disentangle intrinsic dynamics may also facilitate generalization across tasks with different sensory instructions.

A fundamental challenge for nonlinear latent state models is that many alternative models may explain the data equally well. Thus, evaluating learned dynamics relies on computable quantities from measured signals, primarily m-step-ahead neural-behavioral predictions. While our results suggest that in our dataset having nonlinear decoders provides higher performance than other nonlinearities, this might not be case in another dataset. In practice, the optimal nonlinearity can be selected based on the desired metric as done by BRAID. For linear models, all correct latent models have the same eigenvalues (Katayama, 2006), allowing direct evaluation of learned dynamics (figure 2).

## Supplementary Material

Supplement 1

## Figures and Tables

**Table 1:** Related works (see Discussion). ELBO: evidence lower bound, LL: log-likelihood.

Method	Nonlinear	Prioritize behaviorally relevant	Dissociate non-neural	Dissociate intrinsic	Training objective

IPSID	**✗**	**✓**	**✓**	**✓**	Projection-based
TNDM	**✓**	**✓**	**✗**	**✗**	Multi-modal ELBO
LFADS	**✓**	**✗**	**✗**	**✗**	ELBO
mmPLRNN	**✓**	**✗**	**✗**	**✓**	Multi-modal ELBO
DPAD	**✓**	**✓**	**✗**	**✗**	1-step-ahead LL
CEBRA	**✓**	**✓**	**✗**	**✗**	Contrastive
**BRAID**	**✓**	**✓**	**✓**	**✓**	m-step-ahead LL

**Table 2:** 1-step-ahead prediction results for nonlinear simulations with sinusoidal behavior mapping. Mean ± s.e.m. is across 20 runs (10 datasets, 2 folds). State dimension is always set to ground truth. True model’s outcome indicates the “Ideal” accuracy. **Bold**: within 1 s.e.m. of ideal.

Method	Behavior decoding CC	Neural prediction CC

IPSID	0.4567 ± 0.0527	**0.8901 ± 0.0304**
linear BRAID	0.4558 ± 0.0528	**0.8893 ± 0.0304**
DPAD *Nonlin* $C_{z}$	0.4958 ± 0.0620	0.3767 ± 0.0680
BRAID *Nonlin* A	0.4735 ± 0.0572	0.8127 ± 0.0528
BRAID *Nonlin* K	0.7680 ± 0.0467	0.6983 ± 0.0473
BRAID *Nonlin* $C_{y}$	0.4558 ± 0.0528	**0.8887 ± 0.0305**
BRAID *Nonlin* $C_{z}$	**0.8696 ± 0.0487**	**0.8913 ± 0.0305**
BRAID ***Auto*** *Nonlin*	**0.8693 ± 0.0487**	**0.8913 ± 0.0305**

True model (ideal)	0.8737 ± 0.0486	0.8921 ± 0.0306

**Table 3:** Forecasting performance (4-step-ahead) compared to baselines in NHP dataset for models with low $\left(n_{x}=16\right)$ and high-dimensional $\left(n_{x}=64\right)$ latent states. $n_{1}=16$ for BRAID, linear BRAID, and DPAD. $R^{2}$ results were similar (table A.8). Tables A.4, A.5 and A.6 have additional results.

Method	Behavior forecasting CC		Neural forecasting CC	

	$n_{x}=16$	$n_{x}=64$	$n_{x}=16$	$n_{x}=64$

linear BRAID	0.7453 ± 0.0066	0.7409 ± 0.0059	0.1767 ± 0.0054	0.3784 ± 0.0078
DPAD	0.6706 ± 0.0096	0.7352 ± 0.0079	0.2067 ± 0.0062	0.3611 ± 0.0080
U-BRAID	0.7663 ± 0.0069	**0.8049 ± 0.0068**	**0.4089 ± 0.0076**	**0.4185 ± 0.0074**
mmPLRNN	0.6851 ± 0.0143	0.7328 ± 0.00361	0.3162 ± 0.0107	0.3570 ± 0.0223

BRAID (ours)	**0.8042 ± 0.0085**	**0.7970 ± 0.0086**	0.3274 ± 0.0078	**0.4123 ± 0.0077**

## 8 Reproducibility Statement

To ensure the reproducibility of our work, we are sharing the code for BRAID along with a Python notebook demonstrating its usage at https://github.com/ShanechiLab/BRAID. We also provide model architecture and training details in appendix A.1.4. Finally, the dataset we used (O’Doherty et al., 2017) is publicly available for anyone interested in reproducing the results reported in section 4.2.
