# Supplementary material for: BRAID: Input-driven nonlinear dynamical modeling of neural-behavioral data
Source: ArXiv. 2025 Sep 23:arXiv:2509.18627v1. Preprint. [Version 1] (PMC12486053)
Supplement: Supplement 1 [file NIHPP2509.18627v1-supplement-1.pdf]

A APPENDIX

A.1 METHOD DETAILS

A.1.1 TWO-SECTION FORMULATION

In equations 1, 2, and 3, we combined both latent state sections of our model ($\mathbf{x}_k^{(1)}$ and $\mathbf{x}_k^{(2)}$) for simpler exposition. Here, we present the complete two-section formulation. The predictor form part of the model (equation 2) can be written as follows:

$$\begin{cases} \begin{bmatrix} \mathbf{x}_{k+1|k}^{(1)} \\ \mathbf{x}_{k+1|k}^{(2)} \end{bmatrix} &= \begin{bmatrix} \boldsymbol{A}^{(1)}(\mathbf{x}_{k|k-1}^{(1)}) \\ \boldsymbol{A}^{(2)}(\mathbf{x}_{k|k-1}^{(2)}) \end{bmatrix} + \begin{bmatrix} \boldsymbol{K}^{(1)}(\mathbf{y}_k, \mathbf{u}_k) \\ \boldsymbol{K}^{(2)}(\mathbf{y}_k, \mathbf{u}_k, \mathbf{x}_{k|k-1}^{(1)}) \end{bmatrix} \\ \hat{\mathbf{y}}_{k|k-1} &= \boldsymbol{C}_{\mathbf{y}}^{(1)}(\mathbf{x}_{k|k-1}^{(1)}, \mathbf{u}_k) + \boldsymbol{C}_{\mathbf{y}}^{(2)}(\mathbf{x}_{k|k-1}^{(2)}, \mathbf{u}_k) \\ \hat{\mathbf{z}}_{k|k-1} &= \boldsymbol{C}_{\mathbf{z}}^{(1)}(\mathbf{x}_{k|k-1}^{(1)}, \mathbf{u}_k) + \boldsymbol{C}_{\mathbf{z}}^{(2)}(\mathbf{x}_{k|k-1}^{(2)}, \mathbf{u}_k) \end{cases} \tag{A.1}$$

where we have also included the two-section formulation for the prediction of observations as lines 2-3 of the equation. As before, $\mathbf{y}_k \in \mathbb{R}^{n_y}$ and $\mathbf{z}_k \in \mathbb{R}^{n_z}$ are the observed high-dimensional neural activity and behavior respectively while $\mathbf{u}_k \in \mathbb{R}^{n_u}$ represents the measured inputs to the dynamical system. Here, the overall latent state, $\mathbf{x}_k \in \mathbb{R}^{n_x}$, which describes the dynamics underlying the neural-behavioral data, is constructed such that the behaviorally relevant neural dynamics, represented by $\mathbf{x}_k^{(1)} \in \mathbb{R}^{n_1}$, are dissociated from the the irrelevant ones, represented by $\mathbf{x}_k^{(2)} \in \mathbb{R}^{n_x - n_1}$.

The predictor form RNNs in equation A.1 (i.e., *RNN1* and *RNN2*) are complemented by another set of RNNs (i.e., *RNN1*$_{fw}$ and *RNN2*$_{fw}$) that constitute the generative form part of the model (equation 3), and enable m-step-ahead (for $m > 1$) prediction of latent states and neural-behavioral data. The generative RNNs were again shown with a combined latent state in equation 3 for simpler exposition. The following equations show the complete two-section formulation:

$$\begin{cases} \begin{bmatrix} \mathbf{x}_{k+m|k}^{(1)} \\ \mathbf{x}_{k+m|k}^{(2)} \end{bmatrix} &= \begin{bmatrix} \boldsymbol{A}_{fw}^{(1)}(\mathbf{x}_{k+m-1|k}^{(1)}) \\ \boldsymbol{A}_{fw}^{(2)}(\mathbf{x}_{k+m-1|k}^{(2)}) \end{bmatrix} + \begin{bmatrix} \boldsymbol{K}_{fw}^{(1)}(\mathbf{u}_{k+m-1}) \\ \boldsymbol{K}_{fw}^{(2)}(\mathbf{u}_{k+m-1}, \mathbf{x}_{k+m-1|k}^{(1)}) \end{bmatrix} \\ \hat{\mathbf{y}}_{k+m|k} &= \boldsymbol{C}_{\mathbf{y}}^{(1)}(\mathbf{x}_{k+m|k}^{(1)}, \mathbf{u}_{k+m}) + \boldsymbol{C}_{\mathbf{y}}^{(2)}(\mathbf{x}_{k+m|k}^{(2)}, \mathbf{u}_{k+m}) \\ \hat{\mathbf{z}}_{k+m|k} &= \boldsymbol{C}_{\mathbf{z}}^{(1)}(\mathbf{x}_{k+m|k}^{(1)}, \mathbf{u}_{k+m}) + \boldsymbol{C}_{\mathbf{z}}^{(2)}(\mathbf{x}_{k+m|k}^{(2)}, \mathbf{u}_{k+m}) \end{cases} \tag{A.2}$$

where $m > 1$, and $\mathbf{x}_{k+1|k}^{(1)}$ and $\mathbf{x}_{k+1|k}^{(2)}$ (i.e., $m = 1$) are taken from equation A.1. A visualization of how the formulations in equations A.1 and A.2 are connected is provided in figure 1. In equation A.2, we have also included the two-section formulation for the prediction of neural-behavioral observations as lines 2-3 of the equation, showing that applying the same decoders $\boldsymbol{C}_{\mathbf{y}}^{(1)}/\boldsymbol{C}_{\mathbf{y}}^{(2)}$ and $\boldsymbol{C}_{\mathbf{z}}^{(1)}/\boldsymbol{C}_{\mathbf{z}}^{(2)}$ as in equation A.1 to the m-step-ahead predicted latents $\mathbf{x}_{k+m|k}^{(1)}/\mathbf{x}_{k+m|k}^{(2)}$ gives the m-step-ahead predictions of the neural-behavioral data ($\hat{\mathbf{y}}_{k+m|k}$ and $\hat{\mathbf{z}}_{k+m|k}$).

Equations A.1 and A.2 together constitute the two-section formulation of the BRAID model, which consists of 12 transformations in total: $\boldsymbol{A}(\cdot)$, $\boldsymbol{A}_{fw}(\cdot)$, $\boldsymbol{K}(\cdot)$, $\boldsymbol{K}_{fw}(\cdot)$, $\boldsymbol{C}_{\mathbf{z}}(\cdot)$, and $\boldsymbol{C}_{\mathbf{y}}(\cdot)$, each having two sections denoted with the $.^{(1)}$ and $.^{(2)}$ superscripts.

A.1.2 LEARNING ALGORITHM STEPS

In sections A.1.2-A.1.3, we provide detailed formulations of the optimization stages used in BRAID during learning. For simplicity, we explain the optimizations in terms of 1-step ahead predictions, which involve predictor form parameters of the model. Formulations for m-step-ahead predictions, which constitute additional terms in the overall loss (equations 4), are analogous to those provided here, but instead of the predictor form parameters (equation A.1) they engage the generative form parameters (equation A.2).

Note that regardless of what step-ahead predictions were included during training, the learned model can be used to predict the latent state and neural-behavioral data at m-steps ahead for any desired m using equations 2 and 3 (or A.1 and A.2) together. For example, in figure 3, only $[1, 2, 4, 8]$ step ahead predictions are included in the optimization loss (equation 4, but we evaluate the learned models with predictions up to 32-steps ahead.

We develop a two-stage optimization algorithm for learning parameters of the two sections of the BRAID model (equation A.1). We note that the following 2 stages are sequential. This means that parameters associated with behaviorally relevant states ($\mathbf{x}_k^{(1)}$) are fully learned with *RNN1*, then if needed, the remaining parameters corresponding to non-relevant dynamics ($\mathbf{x}_k^{(2)}$) can be learned via *RNN2*. In all the optimizations described below, we use the mean-squared-error (MSE) of predicting observations as the loss function, but we note that the MSE is proportional to the negative log-likelihood (NLL) for isotropic Gaussian-distributed data. Below we provide the details for the 4 optimizations that are performed in the two learning stages of BRAID.

Stage 1:

1a First, BRAID learns a recurrent neural network (*RNN1*) with n_1 states, to minimize behavior prediction MSE given past neural data and inputs (equation A.3). This ensures that *RNN1* only learns neural dynamics that are relevant to (i.e., predictive of) behavior. The states of *RNN1* constitute the first set of latent states in the BRAID model: $\mathbf{x}_k^{(1)}$. This optimization step can be formulated as:

$$\begin{cases} \mathbf{x}_{k+1}^{(1)} & = & \boldsymbol{A}^{(1)}(\mathbf{x}_k^{(1)}) + \boldsymbol{K}^{(1)}(\mathbf{y}_k, \mathbf{u}_k) \\ \mathbf{z}_k & = & \boldsymbol{C}_{\mathbf{z}}^{(1)}(\mathbf{x}_k^{(1)}, \mathbf{u}_k) \\ loss & : & MSE(\mathbf{z}_k, \boldsymbol{C}_{\mathbf{z}}^{(1)}(\mathbf{x}_k^{(1)}, \mathbf{u}_k)) \end{cases} . \tag{A.3}$$

1b Next, in a second optimization, we learn a transformation $\boldsymbol{C}_{\mathbf{y}}^{(1)}(\cdot)$ that maps $\mathbf{x}_k^{(1)}$ to neural activity while minimizing neural prediction MSE (equation A.4):

$$\begin{cases} \mathbf{y}_k & = & \boldsymbol{C}_{\mathbf{y}}^{(1)}(\mathbf{x}_k^{(1)}, \mathbf{u}_k) \\ loss & : & MSE(\mathbf{y}_k, \boldsymbol{C}_{\mathbf{y}}^{(1)}(\mathbf{x}_k^{(1)}, \mathbf{u}_k)) \end{cases} . \tag{A.4}$$

The above 2 steps conclude stage 1 of learning, i.e., learning intrinsic behaviorally relevant dynamics $\mathbf{x}_k^{(1)}$. Next we explain the (optional) remaining stage 2, which can learn any remaining dynamics in neural activity $\mathbf{x}_k^2$.

Stage 2:

2a We learn a second recurrent neural network (*RNN2*) with $n_2 := n_x - n_1$ states, to minimize the MSE loss of predicting the residual neural activity, i.e., $\mathbf{y}'_k := \mathbf{y}_k - \boldsymbol{C}_{\mathbf{y}}^{(1)}(\mathbf{x}_k^{(1)}, \mathbf{u}_k)$, given past neural activity and inputs (equation A.5). States of *RNN2*, i.e., $\mathbf{x}_k^{(2)}$, together with $\mathbf{x}_k^{(1)}$ from stage 1 constitute the full neural dynamics, i.e., $\mathbf{x}_k = \begin{bmatrix} \mathbf{x}_k^{(1)} & \mathbf{x}_k^{(2)} \end{bmatrix}^T$. This optimization step can be formulated as:

$$\begin{cases} \mathbf{x}_{k+1}^{(2)} & = & \boldsymbol{A}^{(2)}(\mathbf{x}_k^{(2)}) + \boldsymbol{K}^{(2)}(\mathbf{y}_k, \mathbf{u}_k, \mathbf{x}_k^{(1)}) \\ \mathbf{y}'_k & = & \boldsymbol{C}_{\mathbf{y}}^{(2)}(\mathbf{x}_k^{(2)}, \mathbf{u}_k) \\ loss & : & MSE(\mathbf{y}'_k, \boldsymbol{C}_{\mathbf{y}}^{(2)}(\mathbf{x}_k^{(2)}, \mathbf{u}_k)) \end{cases} . \tag{A.5}$$

2b Finally, another readout, $\boldsymbol{C}_{\mathbf{z}}^{(2)}$, can be learned to map $\mathbf{x}_k^{(2)}$ to the residual behavior, i.e., $\mathbf{z}'_k := \mathbf{z}_k - \boldsymbol{C}_{\mathbf{z}}^{(1)}(\mathbf{x}_k^{(1)}, \mathbf{u}_k)$, to minimizing the overall behavioral loss (equation A.6):

$$\begin{cases} \mathbf{z}'_k & = & \boldsymbol{C}_{\mathbf{z}}^{(2)}(\mathbf{x}_k^{(2)}, \mathbf{u}_k) \\ loss & : & MSE(\mathbf{z}'_k, \boldsymbol{C}_{\mathbf{z}}^{(2)}(\mathbf{x}_k^{(2)}, \mathbf{u}_k) \end{cases} . \tag{A.6}$$

Note that the optimization in stage 2b does not change *RNN2* or $\mathbf{x}_k^{(2)}$ that were learned in stage 2a. So although stage 2b is supervised by behavior, the second set of states $\mathbf{x}_k^{(2)}$ are still learned unsupervised with respect to behavior.

In case a very low state dimension is specified by the user for stage 1 (n_1 lower than the ground truth shared dimensionality), *RNN1* would not have enough capacity to learn all behaviorally relevant dynamics. In that case, some behaviorally relevant neural dynamics will be left for *RNN2* in stage 2 to learn. This is why the $\boldsymbol{C}_{\mathbf{z}}^{(2)}$ transformation from $\mathbf{x}_k^{(2)}$ to behavior is included in the model, to allow such behaviorally relevant information in $\mathbf{x}_k^{(2)}$ to be utilized to improve behavior decoding.

A.1.3 NON-ENCODED BEHAVIOR-SPECIFIC DYNAMICS

To remove the non-encoded behavior-specific dynamics, as a preprocessing step, we fit a high-dimensional ($n_x = 150$ in all real data analyses) unsupervised RNN to extract neural dynamics alone by minimizing neural prediction MSE (equation A.7):

$$\begin{cases} \mathbf{x}_{k+1}^{(0)} &= \boldsymbol{A}^{(0)}(\mathbf{x}_k^{(0)}) + \boldsymbol{K}^{(0)}(\mathbf{y}_k, \mathbf{u}_k) \\ \mathbf{y}_k &= \boldsymbol{C}_{\mathbf{y}}^{(0)}(\mathbf{x}_k^{(0)}, \mathbf{u}_k) \\ loss &: MSE(\mathbf{y}_k, \boldsymbol{C}_{\mathbf{y}}^{(0)}(\mathbf{x}_k^{(0)}, \mathbf{u}_k)) \end{cases} . \tag{A.7}$$

Then a readout $\boldsymbol{C}_{\mathbf{z}}^{(0)}$ is trained to map these neurally relevant states $\mathbf{x}_k^{(0)}$ to behavior:

$$\begin{cases} \mathbf{z}_k &= \boldsymbol{C}_{\mathbf{z}}^{(0)}(\mathbf{x}_k^{(0)}) \\ loss &: MSE(\mathbf{z}_k, \boldsymbol{C}_{\mathbf{z}}^{(0)}(\mathbf{x}_k^{(0)})) \end{cases} \tag{A.8}$$

After parameters of the above are learned, we run inference on the training data to obtain the filtered behavior as output of the preprocessing RNN model (first line in equation A.8) and subsequently use it in place of the original behavior in BRAID (in equations A.1, A.3 , A.6). In simulations, we validate that this additional stage can successfully remove any input-driven behavior dynamics not encoded in the neural recordings (figure A.2). We include this preprocessing step in all reported real data analyses with BRAID.

Stage 3: As mentioned in 3.3, if desired, BRAID can also learn behavior-specific dynamics as separate dissociated latent states using a post-hoc learning step. This step is performed after BRAID's main learning is done and is meant to be used in conjunction with BRAID's preprocessing stage explained above. In this post-hoc learning step (stage 3), we first infer the behavior using the originally learned BRAID model. We then obtain the residual behavior ($\mathbf{z}_k''$) by subtracting the inferred behavior from the measured behavior. We then learn a third RNN (*RNN3*) that optimizes the prediction of the residual behavior using *only* the external inputs. The following equations summarize this step:

$$\begin{cases} \mathbf{z}_k'' &:= \mathbf{z}_k - [\boldsymbol{C}_{\mathbf{z}}^{(1)}(\mathbf{x}_k^{(1)}, \mathbf{u}_k) - \boldsymbol{C}_{\mathbf{z}}^{(2)}(\mathbf{x}_k^{(2)}, \mathbf{u}_k)] \\ \mathbf{x}_{k+1}^{(3)} &= \boldsymbol{A}^{(3)}(\mathbf{x}_k^{(3)}) + \boldsymbol{K}^{(3)}(\mathbf{u}_k) \\ \mathbf{z}_k'' &= \boldsymbol{C}_{\mathbf{z}}^{(3)}(\mathbf{x}_k^{(3)}, \mathbf{u}_k) \\ loss &: MSE(\mathbf{z}_k'', \boldsymbol{C}_{\mathbf{z}}^{(3)}(\mathbf{x}_k^{(3)}, \mathbf{u}_k)) \end{cases} . \tag{A.9}$$

We summarize BRAID's three stages and their use-case in table A.1.

A.1.4 MODEL ARCHITECTURE DETAILS AND HYPERPARAMETERS

Throughout the manuscript, to model nonlinearities within any of the transformations i.e., $\boldsymbol{A}(\cdot)$, $\boldsymbol{A}_{fw}(\cdot)$, $\boldsymbol{K}(\cdot)$, $\boldsymbol{K}_{fw}(\cdot)$, $\boldsymbol{C}_{\mathbf{z}}(\cdot)$, and $\boldsymbol{C}_{\mathbf{y}}(\cdot)$, we use a multi-layer perceptron (MLP), also known as a feedforward neural network, with a single hidden layer, 64 units in the hidden layer, and a *ReLU* nonlinearity as activation function. Otherwise, to keep a transformation linear, we replace the MLP with a linear mapping implementing a matrix multiplication, which is a special case of an MLP with

Table A.1: Summary of the three stages of BRAID and their use-case in learning various dynamics. Each stage has two RNNs: a predictor form and a generator form RNN, denoted without and with a fw subscript, respectively.

**Stage**	**Models**	**Functionality**
1	*RNN1*, *RNN1*$_{fw}$	Intrinsic behaviorally relevant neural dynamics
2	*RNN2*, *RNN2*$_{fw}$	Residual neural-specific intrinsic dynamics
3	*RNN3*, *RNN3*$_{fw}$	Residual behavior-specific dynamics not encoded in neural activity

no hidden layers and a linear activation function. For example, linear BRAID is a BRAID model with all mappings being linear whereas BRAID *Nonlinear* $\boldsymbol{C}_{\mathbf{z}}$ has a nonlinear MLP as the behavior decoder (both $\boldsymbol{C}_{\mathbf{z}}^{(1)}$ and $\boldsymbol{C}_{\mathbf{z}}^{(2)}$) while all of its other transformations are linear.

We use an Adam optimizer (Kingma & Ba, 2017) in all BRAID optimizations. We train models up to a maximum number of 2500 epochs to ensure convergence, while employing early stopping to avoid overfitting. We provide example learning curves in figure A.7. Details of the hyperparameters used for BRAID are provided in table A.2.

Table A.2: BRAID hyperparameters used in real data experiments and simulations

**Hyperparameter**	**Value**
Number of hidden layers in nonlinear maps	1
Number of hidden units in nonlinear maps	64
Nonlinear activation	ReLU
Learning rate	0.001
Batch size	32
Sequence length	128
Optimizer	Adam

We also show that BRAID can locate the correct structure of nonlinearity within all possible combinations in our simulations (table 2). Here, we set each of the following four groups of transformations i.e., $\boldsymbol{A}(\cdot)/\boldsymbol{A}_{fw}(\cdot)$, $\boldsymbol{K}(\cdot)/\boldsymbol{K}_{fw}(\cdot)$, $\boldsymbol{C}_{\mathbf{y}}(\cdot)$, $\boldsymbol{C}_{\mathbf{z}}(\cdot)$, as linear or nonlinear, resulting in a total of 2^4 cases. To select one final configuration for the nonlinearity, we follow an automatic nonlinearity selection procedure for a given dataset. In this procedure, within the training data, we perform a 2-fold inner cross-validation in which we fit BRAID models with all 2^4 nonlinearity configurations and then pick the nonlinearity structure with the best cross-validated behavior decoding on the held-out section *of the training data*. Then we retrain a BRAID model with that selected structure on the entire training data to get our final model. Finally, we evaluate that final model on the unseen test data. We refer to this approach as automatic nonlinearity selection (table 2).

In simulations, state dimensions are set to be the same as that of the true model underlying the data. In real data analyses, to investigate the effect of n_x, we vary the state dimension in $n_x \in [1, 2, 4, 8, 16, 32, 64]$ and report the results (figure A.5). For BRAID and DPAD models, we always learn the first 16 dimensions via stage 1 i.e., $\mathbf{x}_k^{(1)} \in \mathbb{R}^{n_1}$ with $n_1 = \min(16, n_x)$, and if there is any more capacity left (i.e., if $n_x - n_1 = n_2$ is positive), it is dedicated to the irrelevant states $\mathbf{x}_k^{(2)} \in \mathbb{R}^{n_x - n_1}$ and is learned using stage 2. We pick 16 as the dimensionality of the behaviorally relevant states as in our experiments, because BRAID reached close to its peak behavior decoding at this dimension. We refer to models with state dimensions $n_x = 16$ and 64 as low and high-dimensional regimes, respectively.

A.1.5 Intrinsic dynamics and eigenvalues

To evaluate how well the intrinsic behaviorally relevant neural dynamics are learned, in simulations (figures 2, A.1), we assess the eigenvalues of the generative transition $\boldsymbol{A}_{fw}$ which characterize the intrinsic dynamics. Note that the ground truth and learned models in these simulations both have a linear intrinsic state transition $\boldsymbol{A}_{fw}$ and the nonlinearity either lies in the transformation from latent

states to behavior (figure 2) or transformation from external inputs to the latent space (figure A.1). We learn the forward recursion parameters $\boldsymbol{A}_{fw}$ and $\boldsymbol{K}_{fw}$ of equations 1 and 3 as we optimize multi-step-ahead predictions. We take the eigenvalues of the intrinsic transition $\boldsymbol{A}_{fw}$ and compare them to that of the ground truth model. For the ground truth (λ_i) and identified ($\hat{\lambda}_i$) eigenvalues we first pair them as $\{\lambda_1, \lambda_2, \ldots, \lambda_{n_1}\}$ and $\{\hat{\lambda}_1, \hat{\lambda}_2, \ldots, \hat{\lambda}_{n_1}\}$ such that the sum of squared distances of pairs is minimized. We then calculate the normalized eigenvalue error as:

$$\frac{\sqrt{\sum\limits_{i=1}^{n_1} \|\lambda_i - \hat{\lambda}_i\|^2}}{\sqrt{\sum\limits_{i=1}^{n_1} \|\lambda_i\|^2}}. \tag{A.10}$$

A.2 BASELINES

First, to assess the impact of the nonlinearities learned by BRAID, we compare it against two fully linear dynamical methods: IPSID (Vahidi et al., 2024) and linear BRAID. Second, to highlight the significance of modeling the effect of measured inputs on the neural-behavioral dynamics, we take an autonomous dynamical model, DPAD (Sani et al., 2024), as another baseline. Another important aspect of BRAID is supervision of the behaviorally relevant dynamics in presence of inputs in its first stage. To assess prioritization of the behaviorally relevant dynamics due to this supervision, we take an equivalent unsupervised baseline termed U-BRAID detailed below. We also compare BRAID against a multi-modal nonlinear method that allows accounting for external inputs termed mmPLRNN (Kramer et al., 2022). Finally, we compare BRAID to TNDM (Hurwitz et al., 2021), a second autonomous method for modeling neural-behavioral dynamics.

A.2.1 IPSID

IPSID, similar to BRAID, models the effect of measured external inputs on neural-behavioral dynamics but operates under a fully linear framework. It fits the parameters of a linear version of equation A.1 via a projection-based analytical algorithm called subspace identification (Van Overschee & De Moor, 1996).

A.2.2 LINEAR BRAID

Linear BRAID serves as another linear baseline, retaining the same architecture and learning stages as BRAID but with all transformations replaced by linear mappings. In essence, linear BRAID and IPSID have a similar model that are learned differently. Linear BRAID uses the same numerical optimization used in BRAID. BRAID reduces to linear BRAID by removing all hidden layers within model transformations and setting all activation functions to linear. In simulations, we find that linear BRAID and IPSID perform similarly as expected (figure 2).

A.2.3 DPAD

Dissociative Prioritized Analysis of Dynamics (DPAD) (Sani et al., 2024), learns a nonlinear model that dissociates and prioritizes dynamics shared between neural activity and behavior, but it importantly does not account for the external inputs. Originally, DPAD also does not allow for multi-step-ahead optimization and thus does not learn a generative form representation of the dynamics, which is in contrast to the learning of $\boldsymbol{A}_{fw}$ in BRAID. We extend DPAD to add optimization of multi-step-ahead predictions into the DPAD framework for a more fair comparison to BRAID in terms of forecasting.

A.2.4 U-BRAID

U-BRAID is an unsupervised method, which only performs stage 2 of the BRAID learning procedure. As such, U-BRAID learns all neural dynamics irrespective of their relevance to the behavior, but still while considering inputs (equation A.5). U-BRAID does not utilize behavior information in

learning dynamics, and the extracted latent states are later mapped to the behavior data via a downstream decoder (equation A.6 but without $\mathbf{x}_k^{(1)}$). In fact, U-BRAID is special case of BRAID with $n_1 = 0$ and $n_x = n_2$.

A.2.5 mmPLRNN

Multi-modal piecewise-linear RNN (mmPLRNN) is a method previously introduced for multi-modal dynamical modeling with piecewise-linear RNNs (Kramer et al., 2022). This method allows for modeling external inputs, although this aspect of it has not been investigated in any prior work. Nevertheless, we compare BRAID to an input-driven mmPLRNN to further assess its performance. mmPLRNN builds on a prior work, PLRNN (Durstewitz, 2017), by fusing information from two modalities (e.g., neural activity and behavior). By design, mmPLRNN utilizes both modalities (and input) during inference, which is in contrast to BRAID that only uses neural activity (and input) during inference. Although this provides the benefit of using more data for behavior decoding to mmPLRNN and confounds the comparison with BRAID, we still include the mmPLRNN results. We train mmPLRNN models with nonlinear readouts comparable to BRAID and compare their neural-behavioral forecasting. mmPLRNN is a generative model whose parameters are learned via variational inference. We used the recommended hyperparameters from the original work[1].

A.2.6 TNDM

Targeted Neural Dynamical Modeling (TNDM) (Hurwitz et al., 2021) is a method based on sequential autoencoders that learns two sets of dynamics: one contributing to both neural and behavioral data, and the other only contributing to neural data. TNDM uses a non-causal, bidirectional RNN as the encoder to infer the initial conditions for its relevant and irrelevant generator/decoder RNNs. The dynamical model is learned via variational inference with both neural and behavioral reconstructions optimized simultaneously with a combined loss. Unlike BRAID, TNDM does not account for modeling the effect of external inputs on neural-behavioral dynamics. Therefore, for comparisons, we also implement an extension of TNDM to allow the inclusion of external inputs. In this version, we provide the external inputs to TNDM model as input by concatenating them with the neural activity as the input to the model. Importantly, we do not add reconstruction of inputs as part of the loss to keep the loss the same as that of the original TNDM and keep the learned model focused on neural-behavioral reconstruction.

We compare BRAID to TNDM (with and without addition of sensory stimuli as external input) in our real data experiments. Unlike BRAID that models the neural observations with a Gaussian distribution, TNDM uses a Poisson observation model for the neural data. Therefore, in our comparisons to TNDM, we analyze non-smoothed spike counts in 50ms bins (for both TNDM and BRAID). We use the default hyperparameters from the original work[2] for TNDM.

A.2.7 LFADS

Latent Factor Analysis via Dynamical Systems (LFADS) (Pandarinath et al., 2018) is an unsupervised method that combines nonlinear dynamical modeling with sequential autoencoders. Similar to TNDM, LFADS uses a non-causal, bidirectional RNN as the encoder to infer the initial conditions for a generator/decoder RNN. The dynamical model is learned via variational inference for unsupervised neural reconstruction. LFADS has a version that uses additional RNNs called controller networks to infer unmeasured inputs from the neural data and use those inferred inputs to drive its generator RNN. We use this version of LFADS (O'Shea & Pandarinath, 2021)[3], to serve as an additional benchmark in comparison to BRAID's modeling of measured inputs. For a full visualization of the LFADS model including the controller networks see Supplementary Fig. 12 in Pandarinath et al., 2018. Briefly, without a controller network, all the information about the complete trial has to be encoded into the initial state of the LFADS generator RNN, which then autonomously evolves to extract states and factors over the course of the trial. In contrast, a controller network acts as a regular non-autonomous RNN for LFADS and allows its generator RNN to take corrections from the neural data throughout the trial, hence improving its capacity to accommodate non-smooth changes

[1]We use the implementation provided in `https://github.com/DurstewitzLab/mmPLRNN`

[2]We use the implementation provided in `https://github.com/HennigLab/tndm`

[3]We use the implementation provided in `https://lfads.github.io/lfads-run-manager/`

in the middle of trials (Pandarinath et al., 2018). The controller network itself does not directly take neural data as input, rather an additional bidirectional RNN called the controller-encoder operates on the neural data of each trial, and the states of this controller-encoder are passed as input to the controller network. As a result, the 'inferred inputs' in LFADS are also non-causally inferred. To compare results with BRAID, we set the number of factors, and the latent states of the generator and the controller-encoder networks to be the same as BRAID. We also pass the same smoothed neural data to LFADS and use the Gaussian neural loss option accordingly. Other hyperparameters were set as those in the original work (Pandarinath et al., 2018).

A.2.8 CEBRA

CEBRA (Schneider et al., 2023) is a recent method proposed for extracting latent embeddings from neural data in a way that can be guided by behavior. CEBRA uses a 1-dimensional convolutional network to extract embeddings from small windows of neural data and guides the extraction of latents via a contrastive loss. The supervised version of CEBRA (i.e., CEBRA-Behavior) uses a contrastive loss on behavior to learn embeddings that dissociate samples with different behavior data, and are thus behaviorally relevant. Due to its use of a convolutional network to extract embeddings, CEBRA does not learn explicit recursive dynamics and also extracts embedding from a finite window of neural data at a time. CEBRA also does not learn any models to decode behavior or neural data form the extracted embeddings. Thus, as done in the original work (Schneider et al., 2023), we fit k-NN regression[4] models to decode neural-behavioral data from the extracted CEBRA embeddings. For a fair comparison, we also provided the measured sensory inputs time-series of the task to CEBRA by concatenating them with neural activity as the input. We use the default hyperparameters from the original work[5] for CEBRA.

A.3 Non-human primate electrophysiological recordings from

We analyzed a publicly available dataset (O'Doherty et al., 2017) in which a macaque (monkey I) performs a motor task. Spiking activity was recorded from primary motor cortex (M1), while the subject controlled a 2D cursor to reach targets that appeared on random locations on a grid within a virtual reality environment. Targets appeared back to back, without any time gaps. We took the subject's 2D fingertip position and velocity as the behavior time-series $\mathbf{z}_k$, and the sensory input, taken as 2D location of the current target, as the input signal $\mathbf{u}_k$. We analyzed the first spike dimension available for each channel-resulting in 89 to 92 units from the first 7 available recording sessions and randomly selected half of these units to model as our neural activity. For neural modality, we use spike counts within 50 ms non-overlapping windows. Finally, we smoothed the spike counts by a Gaussian kernel with a 50 ms s.d. (except for in table A.5) and took that as the neural time-series $\mathbf{y}_k$. We report the mean and standard error of the mean (s.e.m.) computed across 7 sessions and 5 cross-validated folds.

As a second neural modality, we also model the raw local field potential (LFP) activity recorded from the same monkey during the same task. As the only preprocessing, we apply a 10 Hz anti-aliasing filter to be able to downsample the raw LFP to the sampling rate of behavior (i.e., 20 Hz). We refer to this modality as raw LFP data. Results for raw LFP data (figure A.3) were consistent with those obtained for spiking activity (figure 3).

A.4 Simulation details

We analyze three simulated datasets based on dynamical systems. In all the three, we generate 10 different sets of random linear matrices for equation A.11, then generate the ground truth latent states $\mathbf{x}_k$, neural activity $\mathbf{y}_k$ and behavior observations $\mathbf{z}_k$. In equation A.11, $\mathbf{w}_k$, $\mathbf{v}_k$, and $\boldsymbol{\epsilon}_k$ are zero-mean white Gaussian noises accounting for unmeasured excitations, neural observation noise, and behavioral observation noise respectively. $f_{C_z}(\cdot)$ and $f_B(\cdot)$ are nonlinear functions, as described below for each simulation.

[4] sklearn.neighbors.KNeighborsRegressor (Pedregosa et al., 2011)

[5] We use the implementation provided in `https://github.com/AdaptiveMotorControlLab/CEBRA`

$$\begin{cases} \mathbf{x}_{k+1} &= \boldsymbol{A}_{fw}\mathbf{x}_k + f_B(\boldsymbol{B}\mathbf{u}_k) + \mathbf{w}_k \\ \mathbf{y}_k &= \boldsymbol{C}_{\mathbf{y}}\mathbf{x}_k + \boldsymbol{D}_{\mathbf{y}}\mathbf{u}_k + \mathbf{v}_k \\ \mathbf{z}_k &= f_{C_z}(\boldsymbol{C}_{\mathbf{z}}\mathbf{x}_k) + \boldsymbol{D}_{\mathbf{z}}\mathbf{u}_k + \boldsymbol{\epsilon}_k \end{cases} \tag{A.11}$$

To generate a temporally structured input in all cases, we simulate a separate random linear state space model according to equation A.12 and take its output as the external input $\mathbf{u}_k$ to the main model of equation A.11.

$$\begin{cases} \mathbf{x}^{\mathbf{u}}_{k+1} &= \boldsymbol{A}^{\mathbf{u}}_{fw}\mathbf{x}^{\mathbf{u}}_k + \mathbf{w}^{\mathbf{u}}_k \\ \mathbf{u}_k &= \boldsymbol{C}_{\mathbf{u}}\mathbf{x}^{\mathbf{u}}_k + \mathbf{v}^{\mathbf{u}}_k \end{cases} \tag{A.12}$$

A.4.1 Simulation 1: Spiral behavior manifold

For the first simulation, we generate data with a spiral behavior manifold. To do so, we apply a pointwise nonlinear mapping $f_{C_z}(\nu) = \left[\frac{\overline{\nu}}{2}\cos(\overline{\nu}) \quad \frac{\overline{\nu}}{2}\sin(\overline{\nu})\right]^T$ to the readout from the latent states (third line in equation A.11). The bar over the function input ν indicates a scaling factor that normalizes it before nonlinear function is applied. See figure 2a for a visualization of the nonlinearity. We take $f_B(\cdot)$ as identity, set dimensions to $n_y = n_z = n_u = n_x = n_1 = 2$, and take $D_{\mathbf{y}} = D_{\mathbf{z}} = 0$ for this simulation.

A.4.2 Simulation 2: Trigonometric behavior manifold

For the second simulation with trigonometric behavior map, we apply another nonlinearity, pointwise sinusoidal nonlinear function, $f_{C_z}(\nu) = a\sin(\overline{\nu}) + b\overline{\nu}$, to the latent states to generate the behavior $\mathbf{z}_k$. See figure 2e for a visualization of the nonlinearity. In this simulation $f_B(\cdot)$ is taken as identity function and we set $n_y = n_z = n_u = n_x = n_1 = 1$.

A.4.3 Simulation 3: Trigonometric input-encoder

For the third simulation, as we iterate over the state equation (first line in equation A.11), we apply a pointwise sinusoidal nonlinear function, $f_B(\nu) = a\sin(\overline{\nu}) + b\overline{\nu}$, to the input. See figure A.1a for a visualization of the nonlinearity. Here $f_{C_z}(\cdot)$ is taken to be an identity function. In this simulation, we set $n_y = n_z = n_u = n_x = n_1 = 1$.

We also perform two additional simulations (figure A.2) that are similar in nonlinearity structure to the second and third simulations explained above, but incorporate an additional 1-dimensional latent state $\mathbf{x}^{(3)}_k$, as in figure 1a, representing input-driven behavior-specific dynamics not encoded in the neural activity, as follows:

$$\begin{cases} \begin{bmatrix}\mathbf{x}^{(1)}_{k+1} \\ \mathbf{x}^{(3)}_{k+1}\end{bmatrix} &= \begin{bmatrix}\boldsymbol{A}^{(1)}_{fw}\mathbf{x}^{(1)}_k \\ \boldsymbol{A}^{(3)}_{fw}\mathbf{x}^{(3)}_k\end{bmatrix} + \begin{bmatrix}f_B(\boldsymbol{B}^{(1)}\mathbf{u}_k) \\ \boldsymbol{B}^{(3)}\mathbf{u}_k\end{bmatrix} + \mathbf{w}_k \\ \mathbf{y}_k &= \boldsymbol{C}^{(1)}_{\mathbf{y}}\mathbf{x}^{(1)}_k + \boldsymbol{D}_{\mathbf{y}}\mathbf{u}_k + \mathbf{v}_k \\ \mathbf{z}_k &= f_{C_z}(\boldsymbol{C}^{(1)}_{\mathbf{z}}\mathbf{x}^{(1)}_k) + \boldsymbol{C}^{(3)}_{\mathbf{z}}\mathbf{x}^{(3)}_k + \boldsymbol{D}_{\mathbf{z}}\mathbf{u}_k + \boldsymbol{\epsilon}_k \end{cases}. \tag{A.13}$$

A.5 Supplementary Results

A.5.1 BRAID can exclude non-encoded behavior-specific dynamics

Here, we demonstrate that the optional preprocessing step in BRAID (detailed in section 3.3) can dissociate behavior-specific dynamics (i.e., those that are not encoded in the neural activity) during learning and make sure they are not conflated with intrinsic neural dynamics and are not mixed into the neural states ($\mathbf{x}^{(1)}_k$ and $\mathbf{x}^{(2)}_k$). We conducted two additional simulations similar in structure to the second and third simulations explained in section 4.1.2. However, here, for all simulated models, we added input-driven dynamics that influenced behavior but were not encoded in neural activity (denoted as $\mathbf{x}^{(3)}_k$ in figure 1a and equation A.13). The preprocessing step is intentionally expected to yield latent states that are potentially less predictive of behavior, but are encoded in neural activity. When desired, BRAID provides the option to further learn behavior-specific dynamics post-hoc

with a separate latent state ($\mathbf{x}_k^{(3)}$). The preprocessing and post-hoc learning steps allow BRAID to avoid conflation of non-encoded behavior dynamics with others, while also being able to learn these dynamics and thus not incurring any overall reduction in behavior decoding.

We fitted BRAID models with the preprocessing, and both with and without post-hoc learning of behavior-specific dynamics. With the preprocessing, BRAID reached the neural prediction performance of the ground truth model indicating correct removal of behavior-specific dynamics (figure A.2). Moreover, the optional learning of behavior-specific dynamics led to reaching the behavior decoding performance of the ground truth model (figure A.2), suggesting that one could optionally learn these dynamics as well within BRAID to gain interpretability (by learning a disentangled model) without compromising decoding performance.

A.5.2 Additional Supplementary Tables

In this section we include additional supplementary tables that further support the results from the main text. The caption for each supplementary table includes all the details, but here we provide a list of these tables:

- Table A.3: Ablation analysis showing the importance of the *RNN*$_{fw}$ generative model in BRAID for learning intrinsic dynamics.
- Table A.4: BRAID results for different nonlinearity configurations in real NHP data.
- Table A.5: Comparison with TNDM in real NHP data.
- Table A.6: Comparison with LFADS and CEBRA in real NHP data.
- Table A.7: BRAID results for modeling different number of neurons in real NHP data.
- Table A.8: Same as table 3 shown in terms of the R^2 metric.

Table A.3: **A separate generative model is essential to learn the intrinsic dynamics accurately with BRAID**.
Row 1: BRAID, when learning a separate generative model (*RNN*$_{fw}$), more accurately learns the intrinsic dynamics as quantified by the error in identifying eigenvalues of the ground truth intrinsic dynamics in the simulated dataset with spiral manifold in figure 2a.
Row 2: The error when ablating the forward RNN from BRAID.
Rows 3-4: DPAD, even when optimized with m-step-ahead prediction loss and/or with input ($\mathbf{u}_k$), does not learn the intrinsic dynamics accurately.

Method	$\log_{10}$ normalized eigenvalue error
BRAID	**-1.3963 ± 0.2551**
BRAID without *RNN*$_{fw}$	0.0635 ± 0.2212
DPAD + m-step loss	0.0357 ± 0.1587
DPAD + input ($\mathbf{u}_k$) + m-step loss	-0.6512 ± 0.0354

Table A.4: Comparison of BRAID model's nonlinearity configurations in the NHP dataset ($n_x = n_1 = 16$, 4-step-ahead).

Model nonlinearity	Behavior forecasting CC	Neural forecasting CC
Linear	0.7453 ± 0.0066	0.1767 ± 0.0054
Recursion ($\boldsymbol{A}, \boldsymbol{A}_{fw}$)	0.7121 ± 0.0059	0.2719 ± 0.0061
Encoder ($\boldsymbol{K}, \boldsymbol{K}_{fw}$)	0.7181 ± 0.0078	0.1646 ± 0.0049
Decoder ($\boldsymbol{C}_{\mathbf{z}}, \boldsymbol{C}_{\mathbf{y}}$)	**0.8042 ± 0.0085**	**0.3274 ± 0.0078**

Table A.5: Comparison to TNDM, both when sensory input is additionally provided to the TNDM model and when it is not (see appendix A.2.6). All models are learned in low-dimensional regime, i.e., BRAID with $n_x = n_1 = 16$, and TNDM with 16 relevant factors only. We used non-smoothed spike counts as the neural signals in this analysis. BRAID performances are for causal 1-step-ahead prediction, whereas the TNDM performances are non-causal smoothing performances, which are the only option for TNDM since it is a sequential autoencoder.

**Method**	**Behavior decoding CC**	**Neural prediction CC**
TNDM	0.3752 ± 0.0170	0.3021 ± 0.0051
TNDM with sensory input	0.6219 ± 0.0103	**0.3075 ± 0.0050**
BRAID (ours)	**0.7841 ± 0.0079**	0.2935 ± 0.0053

Table A.6: Comparison to LFADS (with controller), and CEBRA (CEBRA-behavior). LFADS inferred the external input to the dynamical system (appendix A.2.7). For CEBRA, w.s.i. (with sensory input) indicates that sensory input is additionally provided to the model to obtain the embeddings (appendix A.2.6). All models are learned with both low-dimensional ($n_x = 16$) and high-dimensional ($n_x = 64$) latent states ($n_1 = 16$ for BRAID). BRAID performances are for causal 1-step-ahead prediction, whereas LFADS and CEBRA performances are non-causal smoothing, and 0-step-ahead reconstruction respectively.

**Method**	**Behavior decoding CC**		**Neural prediction CC**	
	$n_x = 16$	$n_x = 64$	$n_x = 16$	$n_x = 64$
LFADS	0.4714 ± 0.0192	0.5891 ± 0.0135	**0.5615 ± 0.0086**	0.5920 ± 0.0072
CEBRA w.s.i.	0.7544 ± 0.0073	0.7514 ± 0.0072	0.5070 ± 0.0053	0.5693 ± 0.0041
BRAID (ours)	**0.8109 ± 0.0074**	**0.8085 ± 0.0076**	**0.5571 ± 0.0051**	**0.8401 ± 0.0061**

Table A.7: **Effect of the number of neurons on BRAID's performance.** Results of the BRAID modeling when different numbers of neurons included as the neural signal. We included neurons from the same channel sets across different sessions and the ranges indicate the number of neurons available from those channels across different sessions. In all 3 rows, neural predictions are evaluated on the same common set neurons (i.e., the smallest set shown in row 1) to make the neural forecasting results comparable across rows. Behavior forecasting improved with more neurons and neural forecasting remained largely stable. These results suggest that BRAID can aggregate behaviorally relevant information across larger populations of neurons, while still being able to model this higher-dimensional population activity well.

**Scale**	**Behavior forecasting CC**		**Neural forecasting CC**	
	$n_x = 16$	$n_x = 64$	$n_x = 16$	$n_x = 64$
20-21 neurons	0.7727 ± 0.0091	0.7709 ± 0.0089	0.3206 ± 0.0081	0.4115 ± 0.0094
41-43 neurons	0.8042 ± 0.0085	0.7970 ± 0.0086	0.3220 ± 0.0088	0.4202 ± 0.0091
89-92 neurons	0.8337 ± 0.0061	0.8302 ± 0.0072	0.3329 ± 0.0082	0.4195 ± 0.0088

Table A.8: R^2 results for the same analyses provided in table 3. Forecasting performance (4-step-ahead) compared to baselines in NHP dataset for models with low-dimensional ($n_x = 16$) and high-dimensional ($n_x = 64$) latent states. $n_1 = 16$ for BRAID, linear BRAID, and DPAD. 1 of the 7 sessions were excluded from this table due to a very negative outlier in the mmPLRNN results.

**Method**	**Behavior forecasting R^2**		**Neural forecasting R^2**	
	$n_x = 16$	$n_x = 64$	$n_x = 16$	$n_x = 64$
linear BRAID	0.5821 ± 0.0056	0.5763 ± 0.0084	0.0239 ± 0.0036	0.1519 ± 0.0067
DPAD	0.4561 ± 0.0141	0.5497 ± 0.0101	0.0321 ± 0.0042	0.1344 ± 0.0069
U-BRAID	0.6047 ± 0.0097	**0.6684 ± 0.0088**	**0.1768 ± 0.0068**	**0.1860 ± 0.0064**
mmPLRNN	0.4737 ± 0.0118	0.6127 ± 0.0369	0.0734 ± 0.0079	0.0563 ± 0.0670
BRAID (ours)	**0.6680 ± 0.0118**	**0.6578 ± 0.0129**	0.1083 ± 0.0060	**0.1792 ± 0.0064**

A.5.3 Additional Supplementary Figures

In this section we include supplementary figures that further support the results from the main text. The caption for each supplementary figure include all the details, but here we provide a list of these supplementary figures:

- Figure A.1: Simulation results for simulation 3 with a trigonometric nonlinear input-encoder.
- Figure A.2: Validation of the optional third stage of learning in BRAID for learning behavior-specific input driven dynamics.
- Figure A.3: Results of modeling the raw LFP modality in the real NHP data.
- Figure A.4: Average low-dimensional latent state trajectory extracted from real NHP data.
- Figure A.5: Results in real NHP data for different latent state dimensions.
- Figure A.6: Example BRAID decoded time series in real NHP data.
- Figure A.7: Example plot showing loss versus epochs for BRAID.

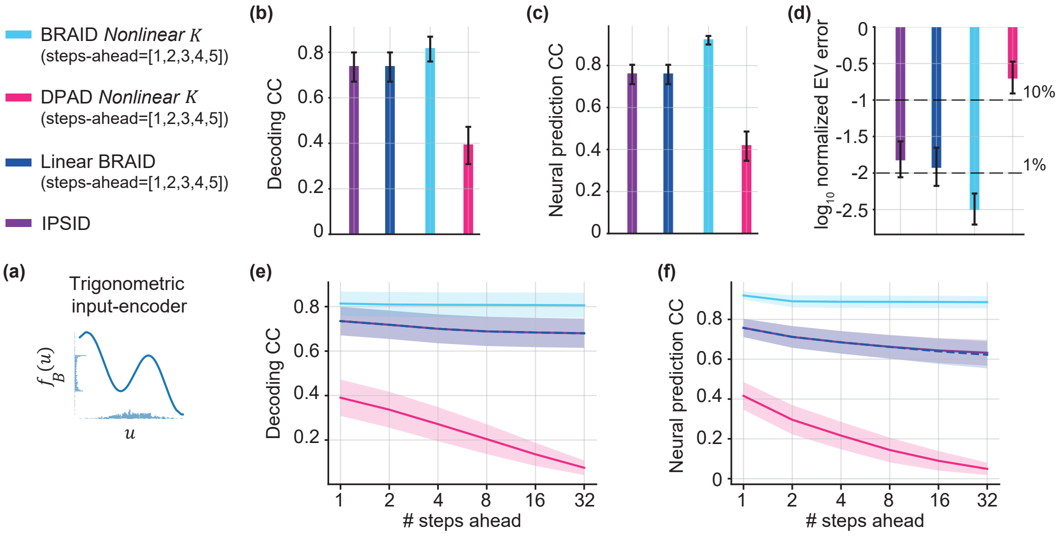

Figure A.1: **BRAID results, optimized for forecasting, in simulation with trigonometric input-encoder.** **(a)** Visualization of example nonlinearity in the simulation. **(b-c)** 1-step-ahead behavior decoding and neural prediction for nonlinear BRAID, nonlinear DPAD, linear BRAID, and IPSID. **(d)** Error in identifying intrinsic dynamics of the true model, quantified by the eigenvalues of the state transition matrix $\boldsymbol{A}_{fw}$. **(e-f)** Behavior and neural forecasting accuracy for 1 to 32 steps ahead, enabled by learning the intrinsic dynamics ($\boldsymbol{A}_{fw}$), with predictions optimized for $[1, 2, 3, 4, 5]$-steps-ahead (section 3.1).

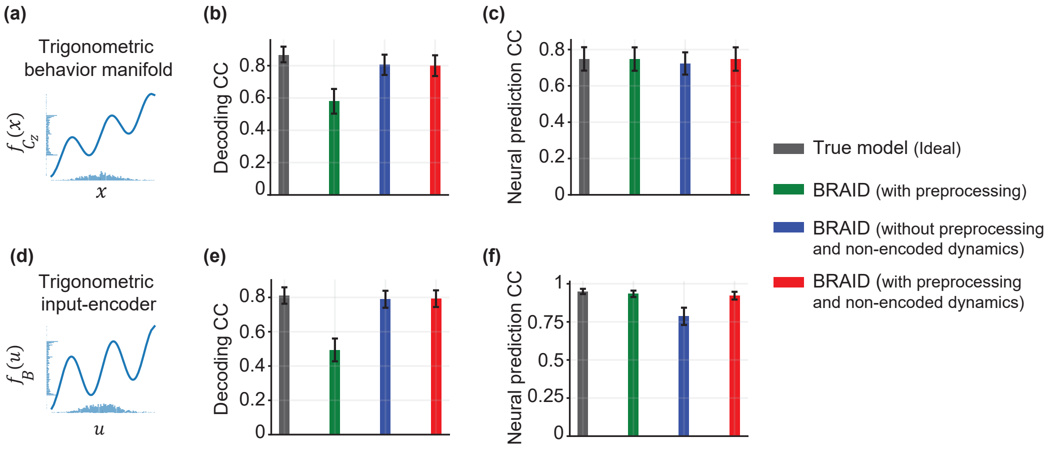

Figure A.2: **Behavior preprocessing successfully excludes non-encoded, behavior-specific dynamics in simulations**. 1-step-ahead behavior decoding and neural predictions for simulations with **(a-c)** trigonometric behavior decoder, and **(d-f)** input-encoder. Note that the true model includes behavior-specific dynamics, so here we expect that decoding of BRAID with preprocessing but without the post-hoc learning of behavior-specific dynamics (shown as green), to be worse than that of true model. Once the post-hoc learning step is also performed (shown as red), BRAID reaches ideal performance, but importantly does so while these behavior-specific dynamics are dissociated into a separate latent state $\boldsymbol{C}_{\mathbf{z}}^{(3)}$. In contrast, without the preprocessing step (shown as blue), BRAID reaches ideal decoding performance, but does so without having dissociated behavior specific dynamics to not be included in $\boldsymbol{C}_{\mathbf{z}}^{(1)}$. See section A.1.3 for details.

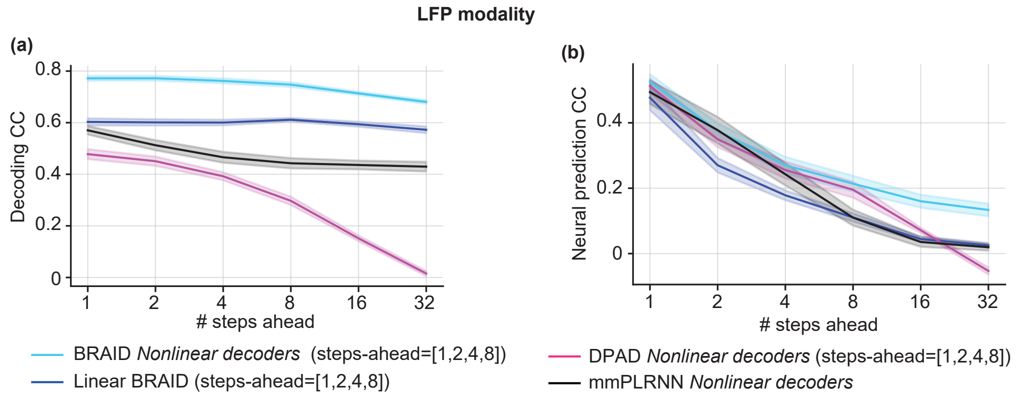

Figure A.3: **Analysis of Local Field Potential (LFP) neural modality. BRAID outperforms baselines in neural-behavioral forecasting**. We used LFP neural data as a second different modality and performed analysis similar to the one in figure 3 for smoothed spike counts. **(a)** Behavior and, **(b)** neural activity forecasting correlation coefficient (CC) for BRAID, linear BRAID, DPAD, and mmPLRNN for n_x=16. Shaded areas show the s.e.m., across the 3 recording sessions and 5 cross-validation folds. NHP results in all other figures and tables are for spiking data.

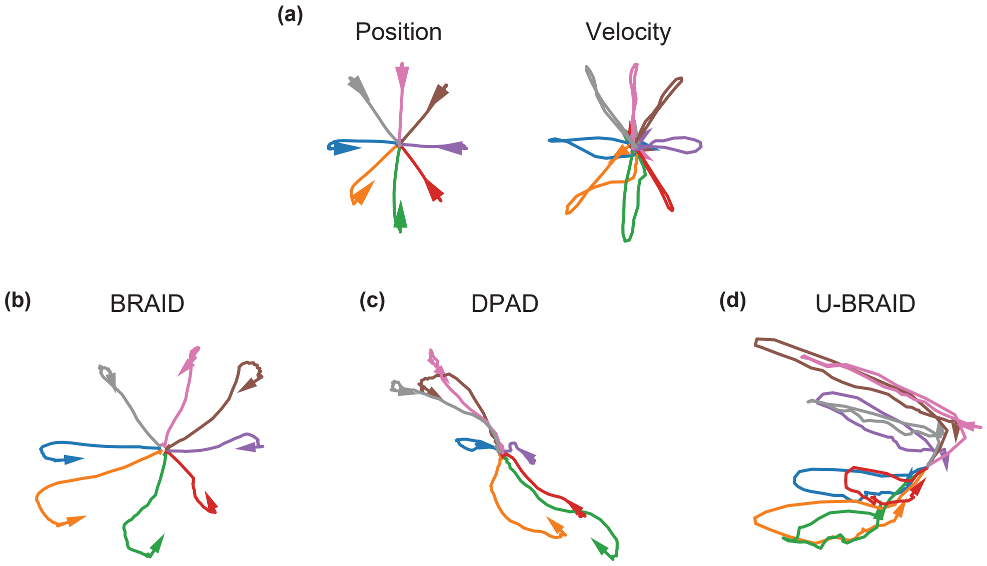

Figure A.4: **Latent states trajectories revealed on non-human primate reaching dataset**. We divide reach trials to 8 conditions based on reach direction (shown by colors) and find the condition-averaged (4-step-ahead) latent states trajectories for 2 dimensional models. **(a)** Condition-averaged behavior i.e., movement position and velocity. **(b-d)** Condition-averaged latent state trajectories for (b) BRAID, (c) DPAD and (d) U-BRAID. BRAID learns the most well-separated posterior (latent) trajectories for the 8 conditions in the task. U-BRAID's trajectories are the least separated, showing that BRAID is more successful in extracting the behaviorally relevant intrinsic dynamics that are more congruent with behavior. Also, DPAD's trajectories are not as well-separated as BRAID's, although they are more separated than the unsupervised version (U-BRAID) as expected.

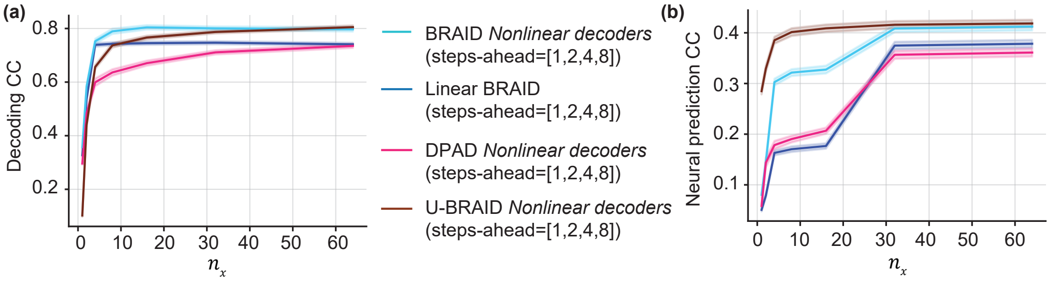

Figure A.5: **Forecasting (4-step-ahead predictions) correlation coefficient across latent dimensions.** BRAID predicts both **(a)** behavior and **(b)** neural activity more accurately than DPAD due to modeling input, and than linear BRAID due to modeling nonlinearity. As state dimension increases beyond 16 (dedicated to behaviorally relevant dynamics, i.e., $n_1 = 16$), BRAID uses stage 2 to learn neural specific dynamics, thus reaching the unsupervised baseline, i.e., U-BRAID, in neural prediction. For BRAID and DPAD, the first 16 state dimensions are dedicated to the behaviorally relevant neural dynamics (i.e., $n_1 = 16$) while any remaining dimensions ($n_x > 16$) are dedicated to the residual non-shared neural dynamics. 4-steps-ahead in this dataset corresponds to 200ms ahead.

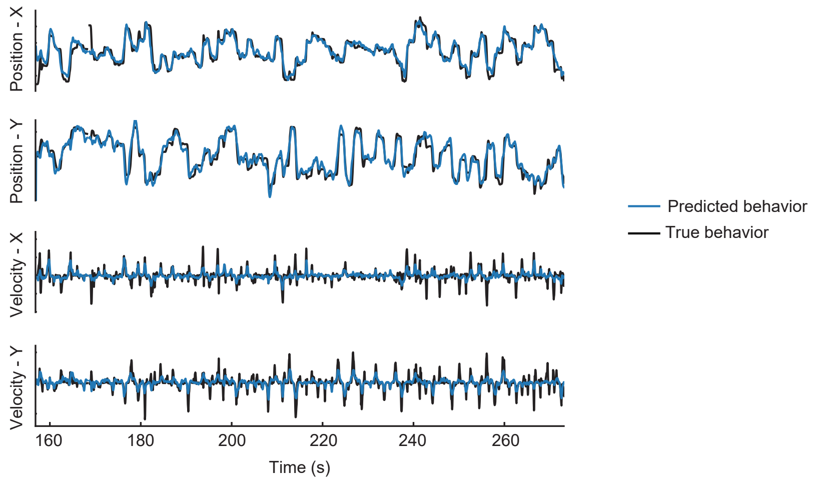

Figure A.6: **Predicted behavior visualization**. True behavior versus BRAID's (4-step-ahead) predicted behavior for a representative session corresponding to the results in table 3 ($n_x = 64$).

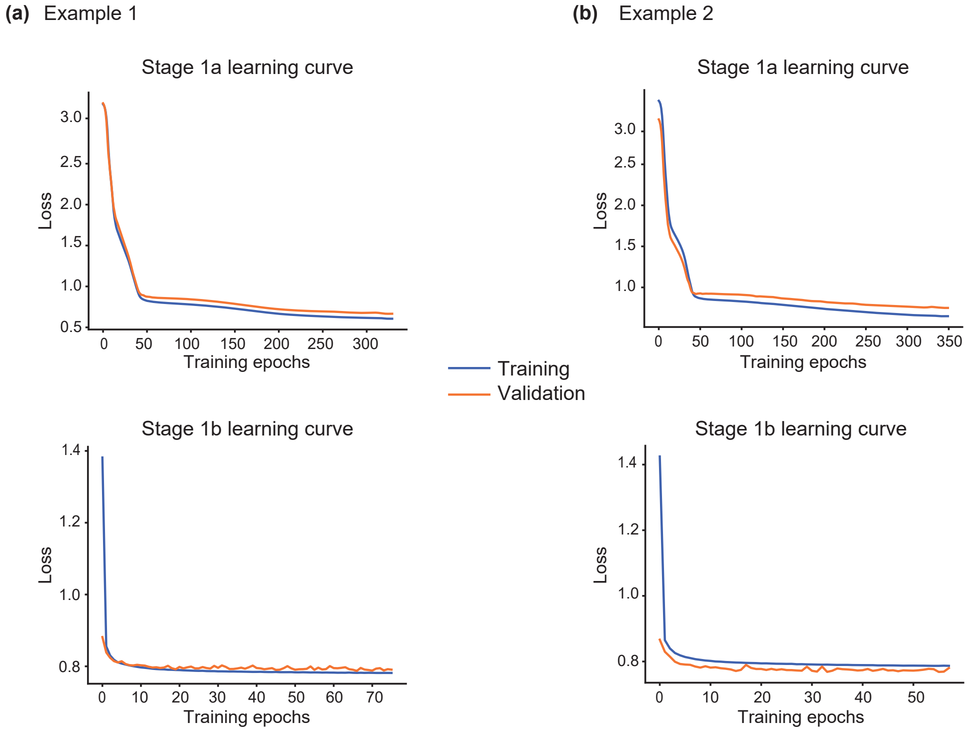

Figure A.7: **Learning curve examples for BRAID model**. Loss values as a function of number of epochs for training and validation datasets for two example runs of BRAID. Top row: learning curve examples for the *RNN1*, *RNN1*$_{fw}$, i.e., stage 1a, in section 3.2. Bottom row: learning curves examples for stage 1b in section 3.2. These examples are taken from the same models whose performances are reported in figure 3 and table 3.
